# Freeform 3D Printing of Cell-Adsorptive Cryogels with Shape-Recoverability

**DOI:** 10.1021/acsmaterialslett.4c01836

**Published:** 2025-02-24

**Authors:** Edgar J. Castanheira, Luís P. G. Monteiro, Rita Sobreiro-Almeida, Vítor M. Gaspar, Nina Kølln Wittig, Tiago R. Correia, Henrik Birkedal, João M. M. Rodrigues, João F. Mano

**Affiliations:** † CICECO—Aveiro Institute of Materials, Department of Chemistry, 56062 University of Aveiro, 3810-193 Aveiro, Portugal; ‡ Department of Chemistry and iNANO, 1006 Aarhus University, Gustav Wieds vej 14, Aarhus 8000, Denmark

## Abstract

The intricate porous network of cryogels enhances diffusivity, injectability, and shape-recovery properties, providing a valuable tool for minimally invasive biomedical applications. However, the injection of cell-loaded complex structures remains highly challenging. Here, we demonstrate the freeform 3D printing of fully protein-based macroporous cryogels with cell-adsorptive and shape-recoverable features. The 3D-printed cryogels surpass conventional cryogels, revealing successful injection, cell-adsorptive features, and a high interconnected porosity (99.2%). The rapid hydration process enables the efficient incorporation of individual cells or spheroids into the cryogel network. Using an in-liquid cell aspiration method, the constructs retained cellular content up to 89%, with *in vitro* culture revealing high viability and cell spreading over 14 days. Our findings highlight the potential of the cryogels’ macroporosity for cell loading and injectability. We anticipate that these off-the-shelf cryogels will pave the way for a new generation of self-adsorptive and injectable cryogels.

## Graphical abstract

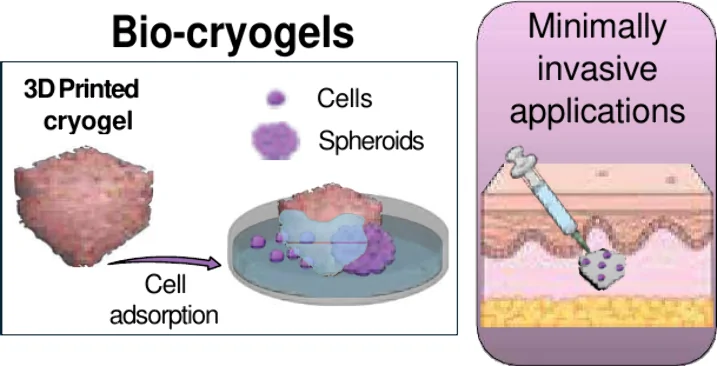

Three-dimensional (3D) porous scaffolds that improve cell adhesion and proliferation are widely explored in the biomedical field to facilitate tissue repair by effectively mimicking *in vivo* cues.^1,2^ Injectable biomaterials are able to fill irregularly shaped defects and establish a proper local microenvironment for tissue healing and regeneration.^3,4^ Macroporous hydrogels formed at subzero temperatures, known as cryogels, have demonstrating tremendous potential as injectable materials in tissue engineering (TE) applications.^5^ The cross-linking of the cryogels at subzero temperatures, followed by thawing, leads to the formation of interconnected macroporous meshes.^6–10^ These structures comprise micro- and macro-interconnected porous networks, which endow cryogels with unique properties: first, the ability to uptake water in an ultrafast manner, holding extra water content within the void spaces;^11^ second, cryogels can withstand high degrees of deformation without rupture, resulting in shape-recoverability upon injection. Furthermore, the porous network creates a physical support for tissue regeneration by facilitating cell migration, proliferation, as well as oxygen and nutrients exchange.^10^ Conventional 3D cryogel scaffolds are often fabricated through shapeable molds.^12–14^ However, additive manufacturing (AM) techniques enables precise fabrication of complex structures.^15,16^

The use of a single element biomaterial ink of natural-based polymers has faced numerous challenges, due to the difficulty in maintaining the filament fidelity or shear-thinning behavior of a low-viscosity ink during extrusion without the introduction of cross-linking agents, fillers, or additional high-viscous materials. For instance, the printability of biodegradable cryogels using methacryloyl gelatin (GelMA) and dialdehyde-functionalized polyurethane as precursors was reported,^13^ nevertheless polyurethane has been correlated with low cell adhesion, biofouling and lack of biostability. The development of cytocompatible 3D-printed GelMA cryogels using an embedded printing technique was also reported.^8^ An ink composed with GelMA, alginate, and a photoinitiator was extruded layer-by-layer into a nanoclay-based support bath. The developed scaffolds were enzymatically degradable but also had an incompatible interaction between GelMA and alginate, which led to phase separation between the layers.

Cryogel biocompatible profile have also relied on common cell culture practices by surface cell seeding,^14,17^ which has been associated with the lack of uniformity of the cell density across the scaffold.^18^ Cell infiltration is directly influenced by the pore network, a factor that is usually neglected.^19^ Although interconnected macroporous networks can enhance the cell infiltration capacity, the effect of cell-laden cryogel injection on cell retention remains unexplored. Herein, we propose freeform 3D-printed cryogels composed solely of GelMA as self-adsorptive/cell-retentive cryogels with exceptional shape-recoverability and cellular retention for minimal invasive applications (Scheme 1).

**Scheme 1:**
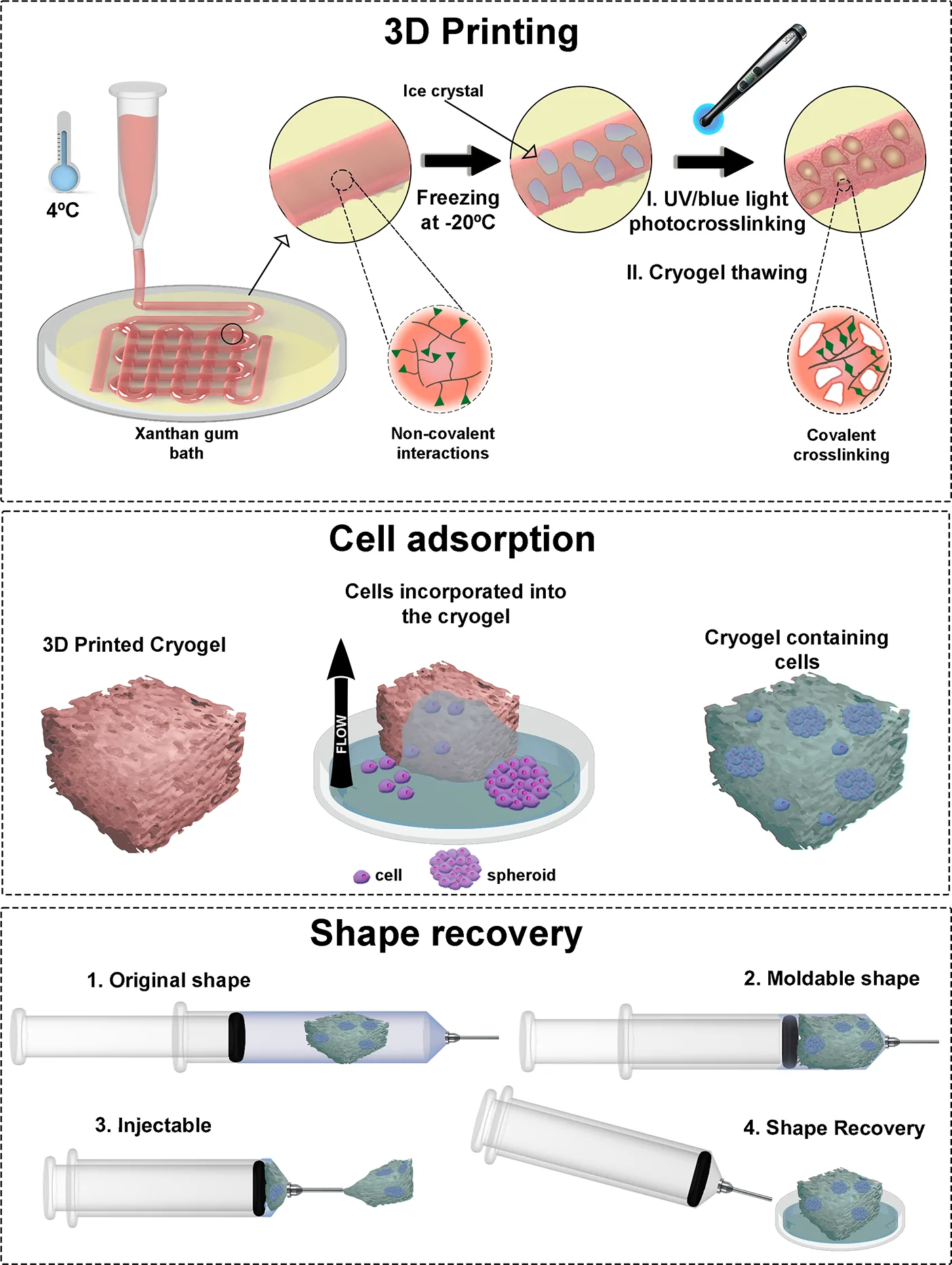
Overall Schematic Representation of the Cryogels Methodology and Properties. Top: Freeform 3D Printing Procedure to Fabricate GelMA Cryogels. Middle: 3D Printed Dried Cryogels Properties and Subsequent Water Uptake of a Cell Suspension Solution Leading to Cellular Adsorption (hASCs and Spheroids). Bottom: 3D Printed Cryogel Injection Showcasing the Deformability and Shape-Recoverability of the Scaffold

GelMA was used as cryogel precursor ink, leveraging its temperature-driven phase transition at 23 °C, enabling its use as a viscous shear-thinning biomaterial ink.^20,21^ Gelatin was first modified with methacryloyl groups, achieving a degree of substitution (DS) of 89% (eq SI 1), confirmed by proton nuclear magnetic resonance (^1^H NMR) analysis (Figure SI1). Three GelMA concentrations, ranging from 1 to 3% (w/v), were assessed to evaluate the most adequate GelMA ink.

Stable gels were obtained only upon irradiation at lower temperatures (Figure 1A). The linear viscoelastic region (LVER) was determined by an amplitude sweep () and a strain value of 0.1% was determined for the photo-cross-linking kinetics at 37 °C (Figure 1B) and 4 °C (Figure 1C). A significant increase in storage modulus (*G*′) upon irradiation was only observed at 4 °C, whereas at 37 °C, *G*′ exhibited constant values for 1% and 2% (w/v) and a slight increment for 3% (w/v) concentration (Figure 1B). ^1^H NMR analysis, performed before and after irradiation, at both temperatures, also confirmed these findings (). The broadening of the bands in the ^1^H NMR spectra at 4 °C indicates a longer relaxation time, suggesting the formation of larger molecules. No broadening occurred at 37 °C, supporting the previous photorheological assays.

**Figure 1:**
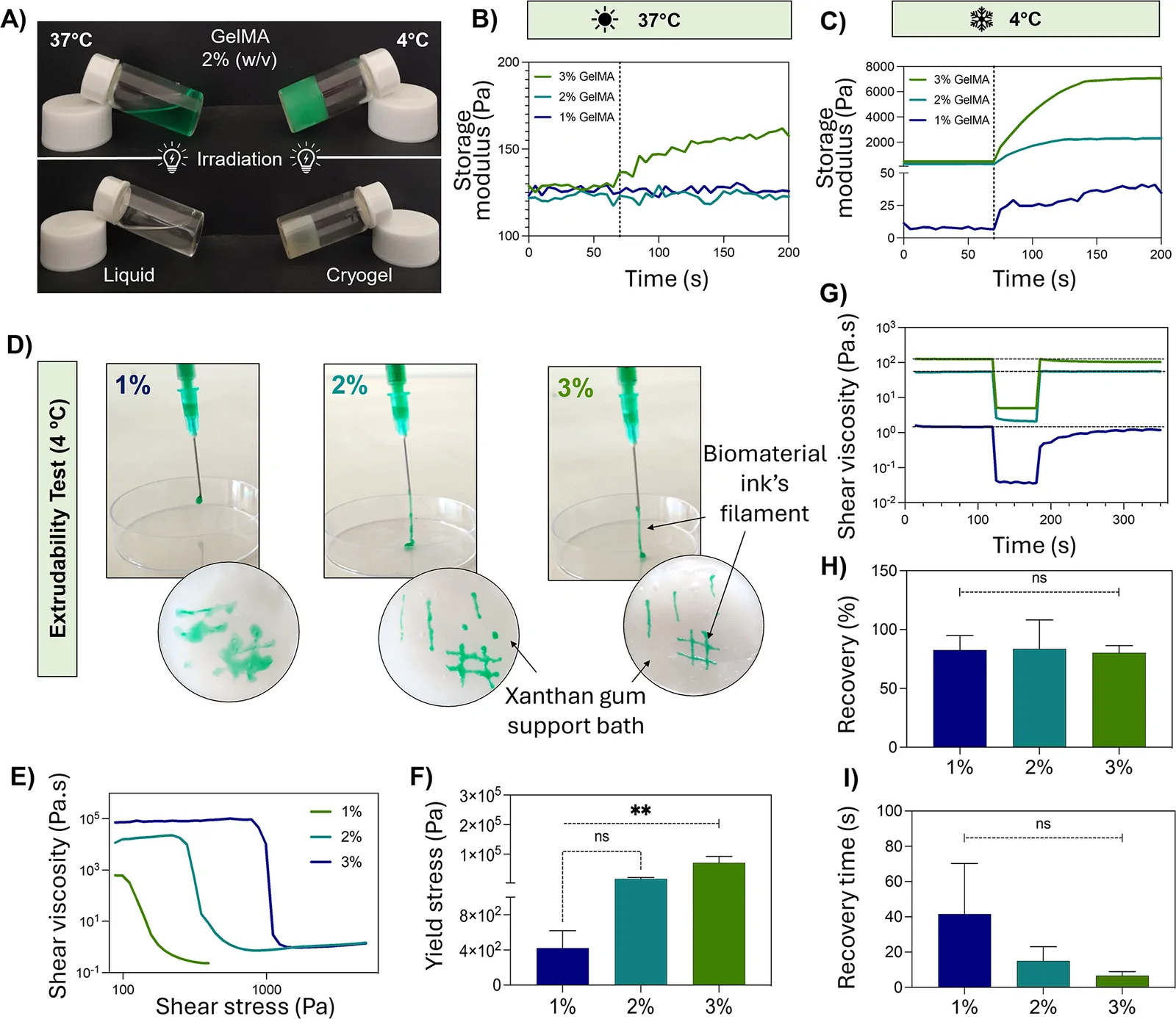
Rheological properties and macroscopic behavior of GelMA biomaterial ink. (A) Photo-cross-linking process before and after irradiation (liquid–hydrogel vs frozen–cryogel). (B) Photorheology of 1%, 2%, 3% (w/v) GelMA biomaterial ink at 37 °C, showing the increment of the storage modulus upon irradiation with a blue light (λ = 395–480 nm). (C) Photorheology of 1%, 2%, 3% (w/v) GelMA biomaterial ink at 4 °C, illustrating the increment of the storage modulus upon irradiation with a blue light (λ = 395–480 nm). (D) Macroscopic evaluation of the 1%, 2%, and 3% (w/v) GelMA biomaterial ink filament extrusion at 4 °C through a 23G needle over a xanthan gum bath. (E) Viscometry rheological assessment flow curves for the 1%, 2%, 3% (w/v) GelMA biomaterial ink at 4 °C. (F) Yield stress extracted from the viscometry graphic. (G) Shear viscosity evolution through a three-stage curve thixotropy for 1%, 2%, 3% (w/v) GelMA biomaterial ink at 4 °C. (H) Recovery (%) of shear viscosity after a high strain. (I) Recovery time (s) of shear viscosity after a hight strain.

Additionally, two flasks containing a 2% (w/v) GelMA solution were exposed to UV-A/blue light: one frozen with liquid nitrogen and the other at room temperature. After irradiation, both samples were subsequently heated to 40 °C. In the first, a cryogel was obtained upon thawing, while for the one at room temperature no hydrogel was obtained (Video SI1). We hypothesize that this phenomenon arises from an entanglement of the triple helical conformation of gelatin at lower temperatures that facilitate the photo-cross-link.^22^

Inks viscoelastic behavior was evaluated for freeform 3D printing extrusion using a xanthan gum (5% (w/v)) as supporting bath.^23^ The different GelMA concentrations were first evaluated by manual extrusion, depositing a filament on the surface of the xanthan gum bath (Figure 1D). The GelMA solution at 1% (w/v) lacked viscosity to hold its shape, while both 2% and 3% (w/v) GelMA inks provided consistent filaments (Video SI2).

Additionally, the viscoelastic properties of GelMA inks were characterized by using a shear stress sweep test (Figure 1E). At low shear stress, a Newtonian plateau was observed, with no changes in the viscosity. As the shear stress increases, the ink behaves as a non-Newtonian fluid, with viscosity decreasing as the shear stress increases.^24^ Moreover, the yield stress describes the minimum shear stress required to initiate the flow (Figure 1F). For 1% (w/v) GelMA ink, the volumetric flow is induced at a shear stress of 15 ± 5 Pa, while 2% and 3% (w/v) the shear stress required was determined to be 299 ± 51 and 742 ± 43 Pa, respectively. Beyond the yield stress point, there is a significant decrease in viscosity, particularly for higher concentrations. Previous studies suggest that higher yield stresses with higher viscosity decay can potentially lead to enhanced printability.^25^

Thixotropic behavior is another factor that significantly influences the ink’s printability (Figure 1G). The shear rate was calculated for a 22G nozzle, at specific 3D printing conditions, based on equations (eqs SI 2 and 3) and on the power-law index (Figure SI4). The different concentrations used had no significant impact on the total recovery (≈85%; Figure 1H). For the rebuilding time, the values ranged from ≈40 s (lower concentration) to ≈6 s (higher concentration) (Figure 1I). Based on the obtained results, the 2% (w/v) GelMA concentration demonstrated to be the most prominent biomaterial ink for freeform 3D printing. This ink revealed the adequate viscosity to achieve a filament with an acceptable yield stress, while showcasing a high recovery value in a considerable short amount of time.^26,27^

To optimize the printing conditions, parameters such as printability factor (*Pr.*) and shape fidelity were evaluated.^28^ Filament fidelity was studied while the printing speed and pressure were varied (Figure SI5A). Lowering the printing speed, filaments with larger diameters were obtained due to a higher volume of extruded material. Conversely, increasing the printing speed reduced the filament width, which is consistent with previous studies.^29^ With printing pressures of 55 and 65 kPa, both filaments exhibited poor fidelity across multiple speeds (Figure SI5B). At a printing pressure of 75 kPa and with the speed at 7 or 10 mm/s, filaments exhibited the highest fidelity.

The pressure was fixed at 75 kPa, and printability was studied at speeds of 7 and 10 mm/s using a square grid mesh (Figure SI5C, D). Visually, 7 mm/s led to mesh structures with better conformation and integrity. The *Pr* value was measured by Equation SI 4.^30^ A perfect square is defined with a *Pr* value equal to one and with 90° inner angles. Hence, for both speeds, high-fidelity 3D-printed cryogel grids were obtained with *Pr.* values of 1.08 ± 0.01 and 1.06 ± 0.02 (Figure SI5E), and with mesh angles of 90.7° ± 13.3 and 89.0° ± 11.2 (Figure SI5F), respectively. However, at 10 mm/s, complex structures lacked integrity to maintain the desired shape during the cryogelation process. Therefore, 75 kPa at a rate of 7 mm/s was used for subsequent studies. Layer stacking accuracy, defined as critical height, was assessed by comparing printed GelMA cryogels with the original design.^28^ The 3D-printed constructs exhibited ≈92% accuracy in critical height, maintaining a high degree of similarity (Figure SI5G). Despite this high fidelity, the printed constructs had a ≈30% increase of surface area compared to the original design (Figure SI5H), which may be explained by the cryogel’s swelling after printing (Figure SI5D). The structural integrity was assessed by compression and decompression of the 3D-printed scaffold with tweezers (Figure SI5I), demonstrating no delamination or rupture.

The mechanical properties of cryogels were also assessed, relying on the photo-cross-link of GelMA. The porosity of the cryogel directly influences its mechanical properties, affecting both injectability and shape recovery. To evaluate the mechanical properties, casted cryogels (C-cryogels) and freeform 3D-printed cryogels (P-cryogels) were prepared (Figure 2A). Unidirectional full compression (Figure 2B, C, D) and cyclic compression (Figure 2E, F, G, H, I) tests were performed. P-cryogels withstood 99% compression without apparent rupture, while C-cryogels ruptured after reaching 55% compressive strain (Figure 2B). At an initial 10% compressive strain, the Young’s modulus was 15.8 ± 5.5 kPa for C-cryogels and 1.7 ± 0.4 kPa for P-cryogels (Figure 2C). The toughness of the C-cryogels was 8.97 ± 1.97 kJ/m^3^, higher than that of the P-cryogels, with 1.69 ± 0.34 kJ/m^3^ (Figure 2D). Since P-cryogels lacked a clear breaking point (Figure 2E), toughness was calculated at 95% of compressive strain.

**Figure 2:**
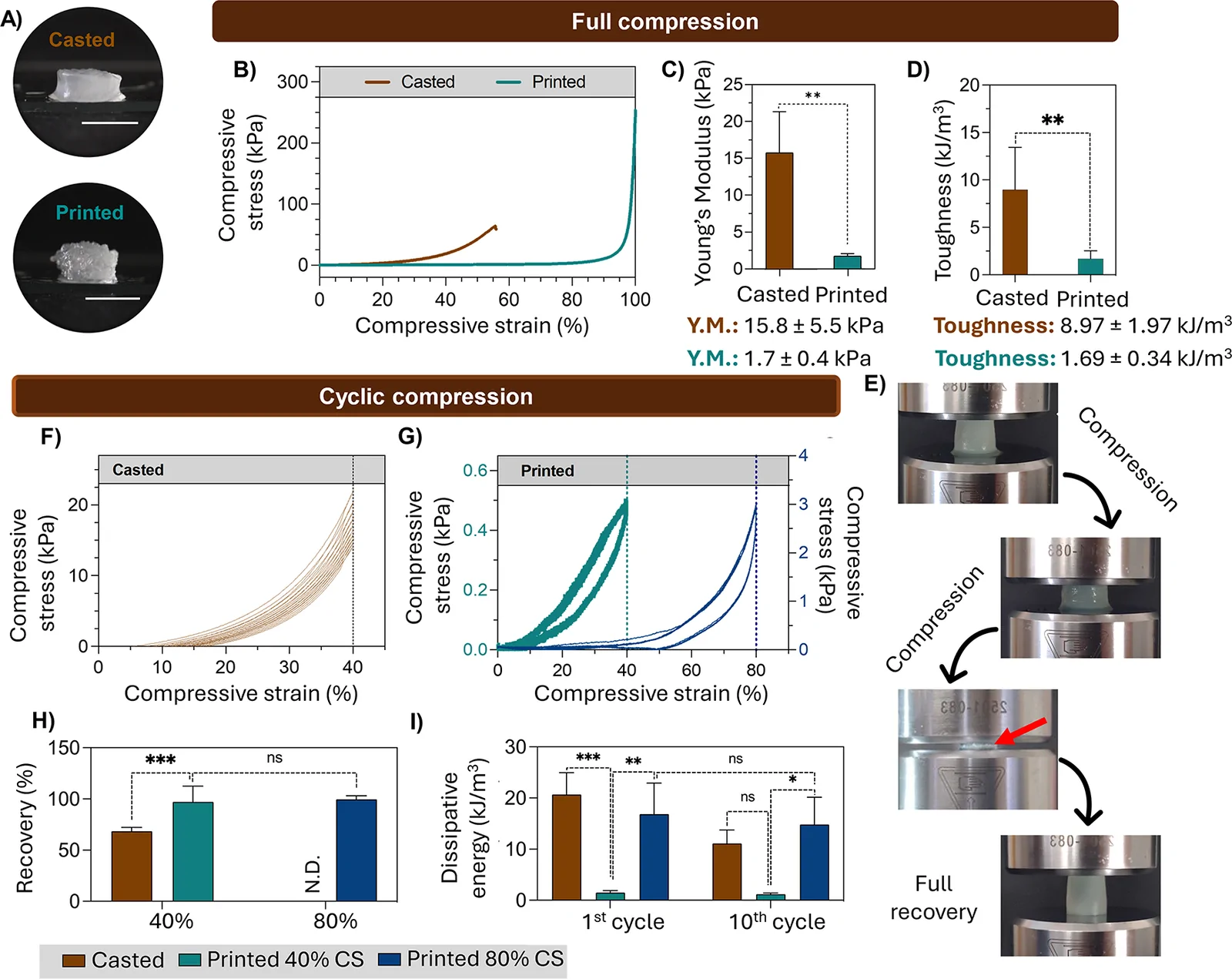
Mechanical properties of C-cryogels and P-cryogels of GelMA at 2% (w/v). (A) Photographic representation showing the visual differences between PDMS C-cryogel and P-cryogel. Scale bar = 5 mm. (B) Unidirectional compressive tests for C-cryogels and P-cryogels until full compression. (C) Young’s modulus and (D) toughness, calculated from unidirectional tests for C-cryogels and P-cryogels. (E) Photographic time frame for the unidirectional compression of P-cryogels, from 0% compressive strain to 80% and back to 0% compressive strain. Scale bar = 5 mm. (F) 10 compressive cyclic stress cycles for the C-cryogel with deformation until 40%. (G) 10 compressive cyclic stress cycles for the P-cryogels at 40% deformation (green) and 80% deformation (blue). (H) Recovery percentage calculated at 40% of deformation for C-cryogels and P-cryogels, and recovery percentage calculated at 80% of deformation for P-cryogels. (I) Dissipation energy at the 1st and 10th cycle for 40% compressive strain for C-cryogel and P-cryogels, and 80% compressive strain for P-cryogels.

With the breaking point determined, cyclic compressive tests were performed at 40% compressive strain for C-cryogels (Figure 2F), and 40% and 80% compressive strain for P-cryogels (Figure 2G). P-cryogels were able to withstand full compression without damage, recovering their original shape upon unloading, demonstrating a high shape-recovery potential (Figure SI6). For C-cryogels, each cycle of loading/unloading affected recoverability, even though the test was conducted at a compressive strain 15% below its breaking point. The recoverability values of P-cryogels were 97 ± 6% and 99 ± 1% for 40% and 80% deformation, respectively. In contrast, for C-cryogels (at 40% compressive strain), the recoverability was only 68 ± 1% (Figure 2H). Lastly, the energy hysteresis (i.e., the ability to dissipate energy through the friction between the pore walls), was calculated. At 40% compressive strain, C-cryogels had a higher dissipative energy (16.9 ± 6.1 kJ/m^3^) when compared with the P-cryogels (1.5 ± 0.4 kJ/m^3^) (Figure 2I). Despite the results for low strains, P-cryogels exhibit high dissipative energy when the compressive strain is increased to 80% (20.7 ± 4.3 kJ/m^3^), indicating excellent self-recovery at higher compressive strains. This result reflects the internal structure of P-cryogels, where the infill density tends to be lower compared to that of C-cryogels. When the P-cryogel is subjected to high compression, a greater number of pore walls interact through friction, squeezing out the water content.^31^ This increased friction leads to enhanced energy dissipation, contributing to the higher dissipative properties observed in 3D-printed cryogels compared with those produced by conventional casting methods.

These cryogels align with existing literature demonstrating a Young’s modulus ranging between 2 and 15 kPa.^8,32,33^ However, comparing their dissipative energy to other 3D-printed cryogels, up to 8-fold increase was observed.^8^ This higher energy dissipation can enhance the structural integrity of the injectable material during delivery and application, making it particularly suitable for dynamic environments such as tissues that undergo movement or mechanical stress. These properties can also influence cell adhesion and proliferation, depending on the cell type used on the scaffolds.^34^ In summary, the mechanical data support that P-cryogels exhibited higher recovery and the ability to withstand higher compressive strain without damage. This insight is crucial for injectability and shape recovery postinjection.

The porous network was also assessed. It is known that freeform 3D printing techniques can induce the formation of macropores, or void spaces, within the porous mesh (Figure 3A). To evaluate the pore size (micro- and macropores) and pore interconnectivity of both types of cryogels, scanning electron microscopy (SEM) equipped with a cooling stage was used (Figure 3B). P-cryogels exhibit a larger pore size, 67 ± 32 μm, while C-cryogels have a size of 27 ± 20 μm (Figure 3C and ).

**Figure 3:**
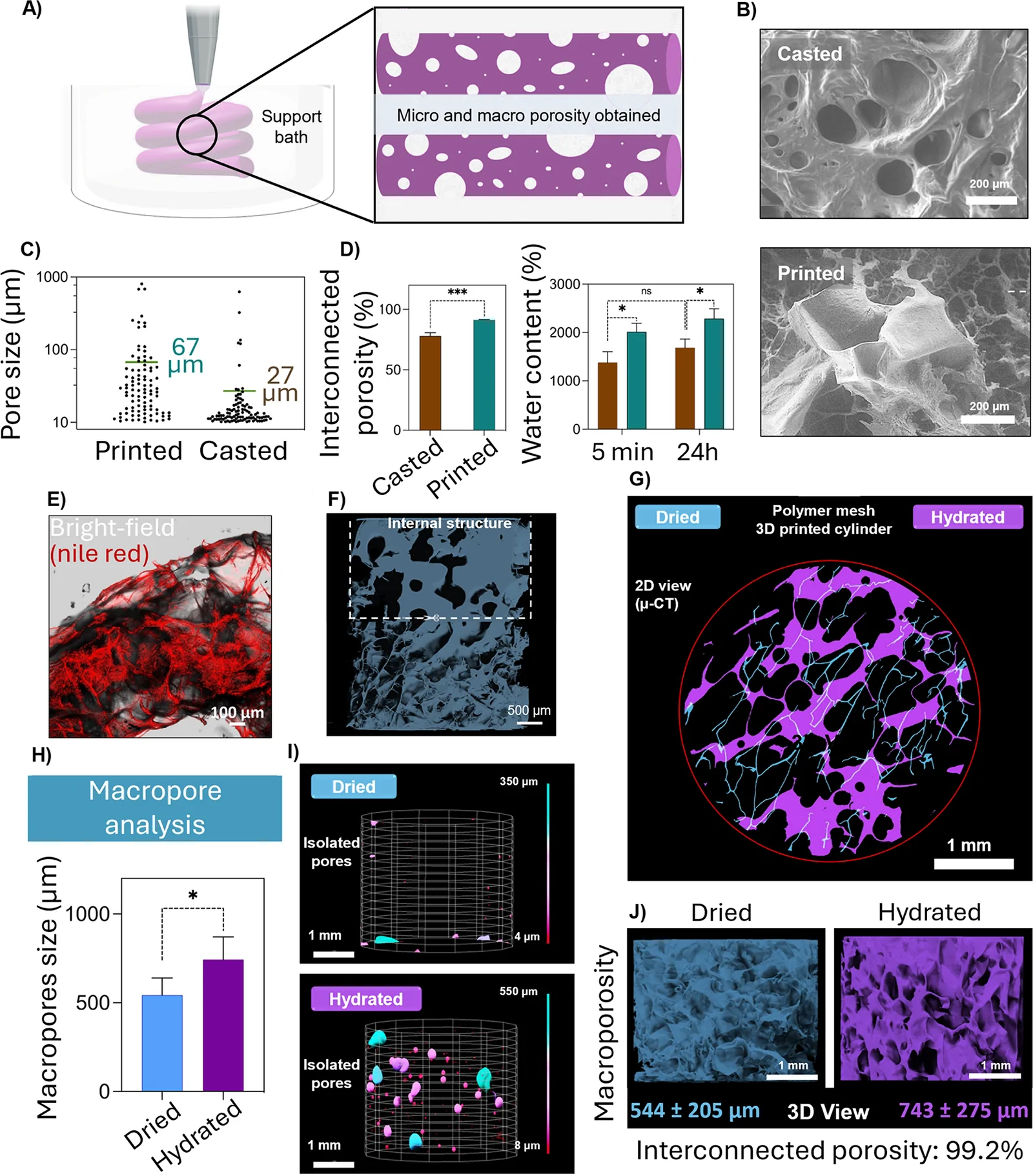
Morphology analysis of C-cryogels and P-cryogels of GelMA at 2% (w/v), and comparison between hydrated and dried forms of the P-cryogels. (A) Schematic representation of the 3D P-cryogels showcasing the micro/macroporosity of the cryogels. (B) SEM images of a cross-section of C-cryogels and P-cryogels. (C) Respective pore analysis of the SEM images. (D) Interconnected porosity percentage (left) and water content of C-cryogels and P-cryogels (right). (E) Confocal image of the P-cryogels stained with alizarin red. (F) X-ray μ-CT image from the surface and inside of the cryogel. (G) Virtual 2D cross section of the segmented/labeled polymer matrix providing an overview of the porous structure between the dried (blue) and hydrated (purple) form of the P-cryogels. (H) Macroporous network and void space size extracted from the 3D images obtained from the μ-CT. (I) 3D μ-CT images of the isolated pores (reverse of the interconnected pores) from the dried (top) and hydrated (bottom) form of the P-cryogels. (J) Cylindrical cropped 3D μ-CT images of the P-cryogels dried (left) and hydrated (right).

Water content (%) was evaluated for both cryogels after immersion in PBS, for 5 min and 24 h (Figure 3D). During the first 5 min, an uptake of approximately 85% of the total water content was observed. Additionally, by quantifying the leaked volume over the total volume it was possible to estimate the interconnected porosity (Figure 3D). P-cryogels presented 91 ± 0.1% interconnected pores, while C-cryogels exhibited only 78 ± 2.6%.

The pore mesh of the P-cryogels was additionally characterized by high-resolution 3D confocal microscopy (Figure 3E) and X-ray microcomputed tomography (μ-CT) (Figure 3F). To compare the features of dried printed (DP-cryogels) and hydrated printed (HP-cryogels) cryogels, a virtual cylinder with the same area was cropped from an inner region of the scaffold. The difference in the micropore size between the dry and hydrated states was assessed (Figure 3G). For the DP-cryogels, the pore size ranged between 63 and 179 μm. For the HP-cryogels, the values ranged from 136 to 219 μm (). Considering the void spaces and the macroporous network (Figure 3H), the pores increased from 544 ± 205 μm (DP-cryogels) to 743 ± 275 μm (HP-cryogels), as supported by the 2D virtual section through 3D μ-CT data sets. Although the scaffold expanded during hydration, the interconnected porosity remained at 99.2% of the total pore content (Figure 3I). Moreover, the 3D printing process was highly homogeneous, with no signs of delamination or collapse of the structure (Figure 3J). The homogeneity of the process is evident across all of the layers, established by the pore wall thickness of the GelMA cryogel (Video SI3). With interconnected porosity exceeding 99%, P-cryogels can be classified as macroporous cryogels with a percolated network of pores.^35^ During the hydration, the pore interconnectivity remained equal while the pore size tended to expand. This creates a potentially adequate environment for cell absorption during cryogels’ hydration.

To assess the structural integrity of the cryogels after injection, the pore structure of P-cryogels were compared before and after injection at 65% compression of their total volume, using C-cryogels as controls (Figure SI9A). No apparent damage to the pore walls or fissures in the protein matrix was observed in the P-cryogels after injection. Additionally, the difference in the pore size before and after injection was not statistically significant, indicating that compression forces of up to 65% did not affect their microstructure. To further evaluate the degradability of the protein matrix, cryogels were incubated at 37 °C in PBS for 14 days (Figure SI9A, B, C) and compared to their initial state (day 0). Although not statistically significant, P-cryogels, whether injected or not, exhibited a tendency for the pore size to decrease after 14 days, suggesting that no pore disruption occurred. Conversely, C-cryogels demonstrated an increase in the pore size over the same period, suggesting higher matrix degradability. These results indicate that the combination of injection and subsequent 14-day degradation had a minimal impact on the P-cryogel structure, which is expected to not significantly influence the overall properties of the scaffolds. These findings were further corroborated by weight loss measurements (Figure SI9D), showcasing that neither injectability nor matrix degradation resulted in statistically significant variations between C-cryogels and P-cryogels.

Subsequently, dried scaffolds were put in contact with suspended human adipose derived stem cells (hASCs). After 5 min of contact, 89 ± 5% of the cells had been adsorbed and adhered to the cryogels (Figure 4A). Osteoblast-like MC3T3-E1 spheroids (diameter ≈500 μm) were also prepared and dispersed in culture medium to assess the scaffold’s capacity to adsorb larger cellular structures, which also resulted in 100% adsorption capacity (Figure 4B, Video SI4). Hence, these cryogels could be used as off-the-shelf natural-based products to create on-demand cell-laden therapeutic solutions.

**Figure 4:**
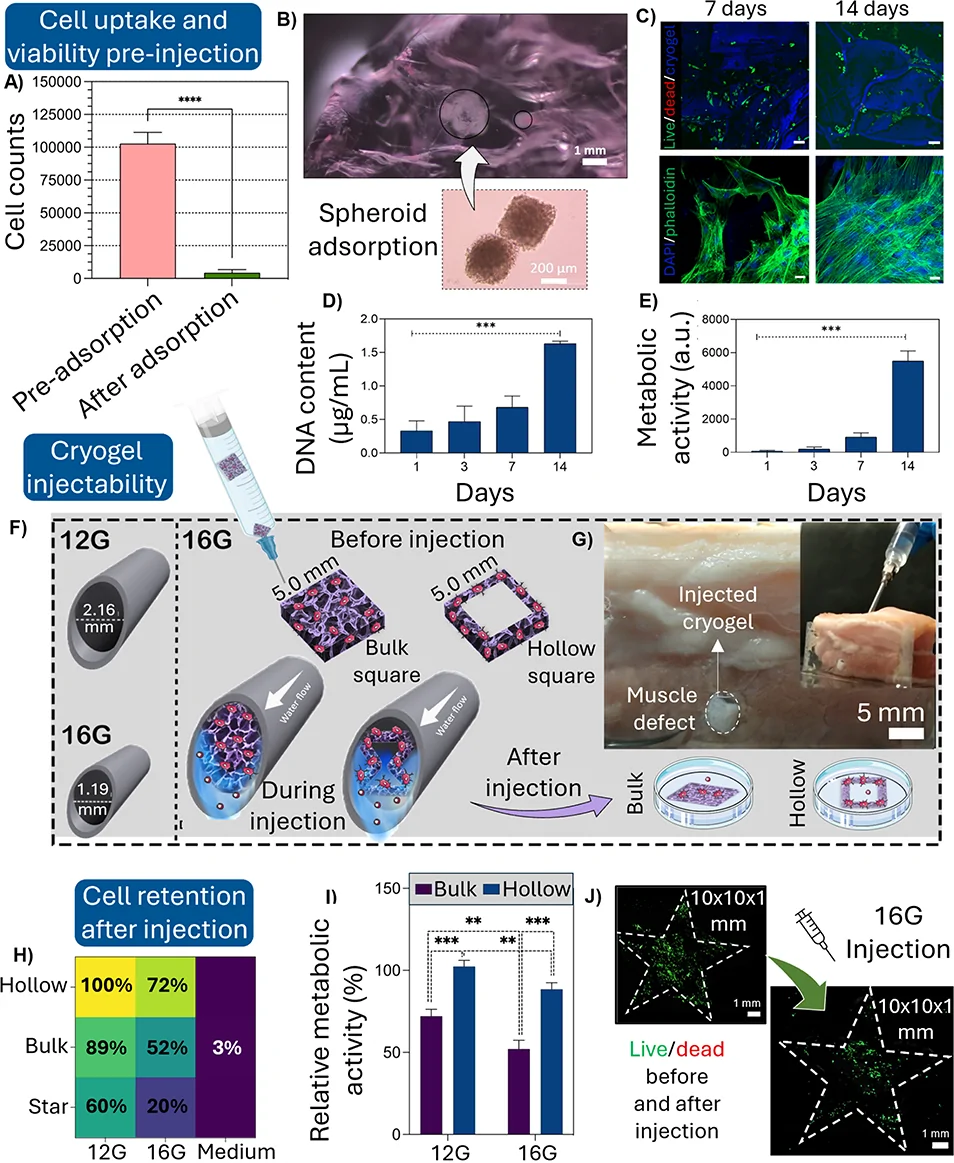
Cells adsorption, cytotoxic assays, and cell retention after injectability of the cell adsorptive cryogels. (A) Flow cytometry of cellular adsorption on cryogels: cell counting plot of hASCs in suspension before (left) and reminiscent in the cell suspension after the GelMA cryogel uptake (right). (B) High cellular adsorption of MC3T3-E1 spheroids (ca. 500 μm) using DP-cryogels. (C) Live–dead images (green: Calcein AM; red: PI) of the P-cryogel incubated with hASCs after 7 and 14 days of culture (top), scale bar = 100 μm, and actin filament (green: phalloidin; blue: DAPI) and nucleus images of the printed cryogel incubated with hASCs after 7 and 14 days of culture (bottom), scale bar = 20 μm. (D) DNA quantification of the P-cryogel incubated with hASCs over 14 days of culture. (E) Metabolic activity (Alamar) quantification of the P-cryogel incubated with hASCs over 14 days of culture. (F) Overview of the injectability methodology of the cell adsorptive P-cryogels; schematic representation of the hollow and bulk square and respective deformation process during injection. (G) Injection of a hollow cylinder cryogel (5 mm) into a pig muscle defect. (H) Heat-map correlating cell retention to shape and gauge after injection using metabolic activity and live/dead assays. (I) Graph plot showcasing the relative metabolic activity of hASCs before and after injection using two different gauges (12G and 16G) and two different scaffolds (bulk and hollow). (J) Cell retention for the star-shaped scaffold (10 × 10 × 1 mm^3^; length × width × height) after cell adsorptive cryogel injection (16G), evaluated through live/dead assay.

Cell viability and metabolic activity of hASCs encapsulated in P-cryogels was evaluated for a 14-day period. Viable and stretched cells were detected on the first days (days 1 and 3), indicating a successful cryogel-mediated cell-adsorption and adherence (Figure SI10). No significant cell death was found during the assay (Figure 4C). Moreover, 3D reconstructed confocal pictures of P-cryogels demonstrated that the cells were homogeneously distributed after just 7 days of culture, which is attributed to the large and interconnected pore network of the cryogels. F-actin filament staining was found to increase from day 7 to day 14, with no apparent damage to the nucleus (Figure 4C).

These findings were also corroborated by other reports, which compared cell migration between cryogels and hydrogels.^34,36^ Additionally, other authors have also reported that even without adhesion motifs like the RGD sequence, the combination of suitable porosity and pore size can still promote cell growth.^34^ Cryogel’s (bio)­degradability, while relevant, plays a smaller role in this context because it is a slow process that does not accelerate early cell adhesion within the matrix.^37^

DNA quantification data showcased the substantial proliferation of the cells on the scaffold (Figure 4D). The normalization of metabolic activity with DNA content demonstrated a progressive increase in the activity of encapsulated hASCs over a period of 14 days (Figure 4E). Both DNA content and cellular metabolic activity indicate that cells were still proliferating within the scaffold by day 14.

Before assessing the cell retention upon injection (Figure 4F), the scaffold was injected into an *ex vivo* premade defect on a pig muscle to confirm the cryogels recovery (Figure 4G). The hollow printed cylinders were injected through 12G and 16G needles, confirming the capability to restore their original shape after deformation (Video SI 5). Following this, we investigated the injection effect on hASCs-laden cryogels after an incubation period of 24 h (Figure 4F). As the cells are subjected to high shear stress, friction, and collision during the compression, a fraction of cells is expected to be lost by death or shedding.^7,38^ Although bulk and hollow scaffolds have similar dimensions, the hollow scaffolds revealed a higher potential to be compressed without causing significant distress to the cells (Figure 4H, I). Our results indicate that cell retention, as well as metabolic activity, was higher on hollow squares with lower syringe gauges, as expected, achieving values of around 102 ± 4%, indicative of low to no shear stress. For the bulk formulations, cell retention was slightly affected and reduced to 72 ± 4%. Likewise, the 16G syringe injection also reduced the cell retention at different levels according to the scaffold structure (89 ± 4% for the hollow and 52 ± 5% for the bulk). In the star-shaped cryogel, due to the dimensions and odd shape, the shear rate and friction increased, resulting in a cell retention of 20 ± 3% (Figure 4H). These results revealed that the infill density and 3D model used during the 3D printing procedure can significantly impact cell retention and, consequently, their viability and metabolic activity. These findings are also corroborated by other reports, where casted GelMA cryogels were injected with a 16G needle, showcasing a 74% of cell retention,^39^ which could be improved by including a sacrificial layer of PEG. In another study, GelMA cryogel microspheres were injected using a 26G syringe, revealing a difference in cell retention of ≈18% (pre- and postinjection), even when using an inner diameter twice the size of the microgels.^38^ In this work, by using a cryogel with a similar or larger size than the needle diameter, we achieved a lower limit of 52% cell retention. Thus, we hypothesize that the 3D printing procedure helps to withstand the shear forces applied during injection.

Herein, it was demonstrated that dried cryogels can be stored and used on-demand to absorb liquid suspensions and, if needed, rapidly encapsulate cells and cellular aggregates. The GelMA 3D-printed cryogels could be proposed, for instance, as on-demand scaffolds for minimally invasive clinical applications by simply resuspending them in a biopsy-isolated suspension of stem cells. Despite requiring one extra step of cell aspiration compared with bioprinting, the number of times cells are exposed to shear stress forces is reduced. Moreover, compared to molding, 3D printing allows for spatial control over the cryogel structure. This is particularly important for applications such as craniofacial spine or joint defects, involving multitissues and interfaces with varying requirements.^40^ These defects, which are usually complex, irregular and difficult to access, require minimally invasive techniques.^41^ In these cases, the strategy proposed herein could provide a robust scaffold structure that press-fits into the precise shape of the defect upon injection. Its cell-instructive interconnected porosity would then allow for new tissue ingrowth and functional multitissue regeneration. Looking ahead, these cryogels could be developed into multilayer scaffolds with a mineralized or composite bottom layer for bone regeneration and a top layer promoting chondrogenesis, better mimicking natural tissues.^42^ However, scaling up the extrusion-based 3D printing process presents challenges for larger structures. The time-consuming nature of this method, along with the difficulties in injecting large constructs, limits its practicality for treating large tissue defects. Alternatively, other 3D printing techniques, such as digital light processing (DLP), could be adapted to enhance the production speed and enable the fabrication of intricate, gravity-defying structures with higher resolution.

The growing demand for off-the-shelf injectable material has increased to facilitate noninvasive techniques. The injectability of shape-recovery 3D-printed materials allows for the fabrication of intricate designs to fill different defects in damaged tissues. These 3D constructs should withstand shear forces, deform, and recover their shape, while also maintaining the integrity of the cellular component preadsorbed to the biomaterial. We reported a GelMA biomaterial ink to fabricate 3D-printed cryogels *via* freeform 3D printing. Due to the interconnected macroporous architecture of cryogels, a new on-demand method to adsorb cells and large cellular aggregates was confirmed, revealing an ultrafast (ca. 4 s) and efficient cell-adsorptive capacity (≥89%). Moreover, printing the cryogels improved the porous structure, demonstrating outstanding injectable performance, while retaining most of the cell content. The freeform printed cryogels offer several advantages: (i) exclusively protein-based composition; (ii) patient-specific personalization of defect site; (iii) prompt to easily adsorb human cells on-demand; and (iv) delivery via minimal invasive techniques due to excellent shape recovery. Additional studies on the influence of initial cellular density and scaffold maturation are envisioned. We hypothesize that cell density could be increased before injection by *ex-vivo* culturing of the scaffold or by increasing the initial cell concentration to be adsorbed. *In vitro* results suggest a strong potential to explore this technology for *in vivo* applications. This work highlights the need for injectable, cell-supportive cryogels with programmable architectural features such as an interconnected macroporous network and high cell adsorption capacity, leveraging a fast and efficient water-uptake mechanism. These materials have shown promising results for treating craniofacial defects, as they can be injected through minimally invasive techniques and withstand multiple compression cycles.

## Supplementary Material













## Notes

Video showing photo-cross-linking (Video SI1) (MP4)
Video showing ink extrusion (Video SI2) (MOV)
Video showing thickness mesh (Video SI3) (MOV)
Video showing spheroids adsorption (MOV)
Video showing cryogel injection (Video SI5) (MOV)
Materials, experimental procedures for GelMA synthesis, ^1^H NMR spectra, rheological and mechanical properties, ink formulations, linear viscoelastic region, 3D printing patterns, cryogels pore size, microscopy, degradability assays, biological assays, and statistical analysis (PDF)

## References

[1] Costa P. D. C., Costa D. C. S., Correia T. R., Gaspar V. M., Mano J. F. (2021). Natural Origin Biomaterials for 4D Bioprinting Tissue-Like Constructs. Advanced Materials Technologies.

[2] Nikolova M. P., Chavali M. S. (2019). Recent advances in biomaterials for 3D scaffolds: A review. Bioact Mater..

[3] Bertsch P., Diba M., Mooney D. J., Leeuwenburgh S. C. G. (2023). Self-Healing Injectable Hydrogels for Tissue Regeneration. Chem. Rev..

[4] Chen J., Yang J., Wang L., Zhang X., Heng B. C., Wang D. A., Ge Z. (2021). Modified hyaluronic acid hydrogels with chemical groups that facilitate adhesion to host tissues enhance cartilage regeneration. Bioact Mater..

[5] Eggermont L. J., Rogers Z. J., Colombani T., Memic A., Bencherif S. A. (2020). Injectable Cryogels for Biomedical Applications. Trends Biotechnol.

[6] Cheng Q. P., Hsu S. H. (2023). A self-healing hydrogel and injectable cryogel of gelatin methacryloyl-polyurethane double network for 3D printing. Acta Biomater.

[7] Wang Y., Yuan Z., Pang Y., Zhang D., Li G., Zhang X., Yu Y., Yang X., Cai Q. (2023). Injectable, High Specific Surface Area Cryogel Microscaffolds Integrated with Osteoinductive Bioceramic Fibers for Enhanced Bone Regeneration. ACS Appl. Mater. Interfaces.

[8] Bilici C., Altunbek M., Afghah F., Tatar A. G., Koc B. (2023). Embedded 3D Printing of Cryogel-Based Scaffolds. ACS Biomater Sci. Eng..

[9] Bencherif S. A., Warren Sands R., Ali O. A., Li W. A., Lewin S. A., Braschler T. M., Shih T. Y., Verbeke C. S., Bhatta D., Dranoff G. (2015). Injectable cryogel-based whole-cell cancer vaccines. Nat. Commun..

[10] Bencherif S. A., Sands R. W., Bhatta D., Arany P., Verbeke C. S., Edwards D. A., Mooney D. J. (2012). Injectable preformed scaffolds with shape-memory properties. Proc. Natl. Acad. Sci. U. S. A..

[11] Li M., Dai Q., Zhu S., Feng Q., Qin Z., Gao H., Cao X. (2023). An ultrafast water absorption composite cryogel containing Iron-doped bioactive glass with rapid hemostatic ability for non-compressible and coagulopathic bleeding. Chem. Eng. J..

[12] Chen T. C., Wong C. W., Hsu S. H. (2022). Three-dimensional printing of chitosan cryogel as injectable and shape recoverable scaffolds. Carbohydr. Polym..

[13] Li M., Zhang Z., Liang Y., He J., Guo B. (2020). Multifunctional Tissue-Adhesive Cryogel Wound Dressing for Rapid Nonpressing Surface Hemorrhage and Wound Repair. ACS Appl. Mater. Interfaces.

[14] Zhao X., Guo B., Wu H., Liang Y., Ma P. X. (2018). Injectable antibacterial conductive nanocomposite cryogels with rapid shape recovery for noncompressible hemorrhage and wound healing. Nat. Commun..

[15] Mirzaali M. J., Moosabeiki V., Rajaai S. M., Zhou J., Zadpoor A. A. (2022). Additive Manufacturing of Biomaterials-Design Principles and Their Implementation. Materials (Basel).

[16] Murphy S. V., Atala A. (2014). 3D bioprinting of tissues and organs. Nat. Biotechnol..

[17] Tripathi A., Kathuria N., Kumar A. (2009). Elastic and macroporous agarose–gelatin cryogels with isotropic and anisotropic porosity for tissue engineering. J. Biomed. Mater. Res., Part A.

[18] Collon K., Bell J. A., Chang S. W., Gallo M. C., Sugiyama O., Marks C., Lieberman J. R. (2022). Effects of cell seeding technique and cell density on BMP-2 production in transduced human mesenchymal stem cells. J. Biomed Mater. Res. A.

[19] Murphy C. M., O’Brien F. J. (2010). Understanding the effect of mean pore size on cell activity in collagen-glycosaminoglycan scaffolds. Cell Adh Migr.

[20] Kurian A. G., Singh R. K., Patel K. D., Lee J. H., Kim H. W. (2022). Multifunctional GelMA platforms with nanomaterials for advanced tissue therapeutics. Bioact Mater..

[21] Kang J. I., Park K. M. (2021). Advances in gelatin-based hydrogels for wound management. J. Mater. Chem. B.

[22] Bigi A., Panzavolta S., Rubini K. (2004). Relationship between triple-helix content and mechanical properties of gelatin films. Biomaterials.

[23] Patricio S. G., Sousa L. R., Correia T. R., Gaspar V. M., Pires L. S., Luis J. L., Oliveira J. M., Mano J. F. (2020). Freeform 3D printing using a continuous viscoelastic supporting matrix. Biofabrication.

[24] Hafeez S., Aldana A. A., Duimel H., Ruiter F. A. A., Decarli M. C., Lapointe V., van Blitterswijk C., Moroni L., Baker M. B. (2023). Molecular Tuning of a Benzene-1,3,5-Tricarboxamide Supramolecular Fibrous Hydrogel Enables Control over Viscoelasticity and Creates Tunable ECM-Mimetic Hydrogels and Bioinks. Adv. Mater..

[25] Mouser V. H., Melchels F. P., Visser J., Dhert W. J., Gawlitta D., Malda J. (2016). Yield stress determines bioprintability of hydrogels based on gelatin-methacryloyl and gellan gum for cartilage bioprinting. Biofabrication.

[26] Chen M., Yang L., Zheng Y., Huang Y., Li L., Zhao P., Wang S., Lu L., Cheng X. (2020). Yield stress and thixotropy control of 3D-printed calcium sulfoaluminate cement composites with metakaolin related to structural build-up. Construction and Building Materials.

[27] Herrada-Manchon H., Fernandez M. A., Aguilar E. (2023). Essential Guide to Hydrogel Rheology in Extrusion 3D Printing: How to Measure It and Why It Matters?. Gels.

[28] Schwab A., Levato R., D’Este M., Piluso S., Eglin D., Malda J. (2020). Printability and Shape Fidelity of Bioinks in 3D Bioprinting. Chem. Rev..

[29] Sobreiro-Almeida R., Gomez-Florit M., Quinteira R., Reis R. L., Gomes M. E., Neves N. M. (2021). Decellularized kidney extracellular matrix bioinks recapitulate renal 3D microenvironment in vitro. Biofabrication.

[30] Ouyang L., Armstrong J. P. K., Lin Y., Wojciechowski J. P., Lee-Reeves C., Hachim D., Zhou K., Burdick J. A., Stevens M. M. (2020). Expanding and optimizing 3D bioprinting capabilities using complementary network bioinks. Science Advances.

[31] Yetiskin B., Okay O. (2019). High-strength and self-recoverable silk fibroin cryogels with anisotropic swelling and mechanical properties. Int. J. Biol. Macromol..

[32] Rana D., Colombani T., Saleh B., Mohammed H. S., Annabi N., Bencherif S. A. (2023). Engineering injectable, biocompatible, and highly elastic bioadhesive cryogels. Mater. Today Bio.

[33] Jonidi Shariatzadeh F., Solouk A., Mirzadeh H., Bonakdar S., Sadeghi D., Khoulenjani S. B. (2024). Cellulose nanocrystals-reinforced dual crosslinked double network GelMA/hyaluronic acid injectable nanocomposite cryogels with improved mechanical properties for cartilage tissue regeneration. J. Biomed Mater. Res. B Appl. Biomater.

[34] Backer A., Goppert B., Sturm S., Abaffy P., Sollich T., Gruhl F. J. (2016). Impact of adjustable cryogel properties on the performance of prostate cancer cells in 3D. Springerplus.

[35] Foudazi R., Zowada R., Manas-Zloczower I., Feke D. L. (2023). Porous Hydrogels: Present Challenges and Future Opportunities. Langmuir.

[36] Hwang Y., Zhang C., Varghese S. (2010). Poly­(ethylene glycol) cryogels as potential cell scaffolds: effect of polymerization conditions on cryogel microstructure and properties. J. Mater. Chem..

[37] Lin T. W., Hsu S. H. (2020). Self-Healing Hydrogels and Cryogels from Biodegradable Polyurethane Nanoparticle Crosslinked Chitosan. Adv. Sci. (Weinh).

[38] Yuan Z., Yuan X., Zhao Y., Cai Q., Wang Y., Luo R., Yu S., Wang Y., Han J., Ge L. (2021). Injectable GelMA Cryogel Microspheres for Modularized Cell Delivery and Potential Vascularized Bone Regeneration. Small.

[39] Dalal N., Challa R., Thimukonda J. J., Tayalia P. (2024). Gelatin Methacryloyl Based Injectable Cryogels with Tunable Degradability for Cell Delivery. Macromol. Biosci.

[40] Kretlow J. D., Young S., Klouda L., Wong M., Mikos A. G. (2009). Injectable biomaterials for regenerating complex craniofacial tissues. Adv. Mater..

[41] Jimson, S. Residual Deformities of the Maxillofacial Region. In Oral and Maxillofacial Surgery for the Clinician; Bonanthaya, K. , Panneerselvam, E. , Manuel, S. , Kumar, V. V. , Rai, A. , Eds.; Springer Nature: Singapore, 2021; pp 1303–1339.

[42] Zhong Y., Cao X., Huang M., Lei Y., Liu A. L. (2025). Biomimetic bone cartilage scaffolds based on trilayer methacrylated hydroxyapatite/GelMA composites for full-thickness osteochondral regeneration. Int. J. Biol. Macromol..

