# Supplementary material for: Freeform 3D Printing of Cell-Adsorptive Cryogels with Shape-Recoverability
Source: ACS Mater Lett. 2025 Feb 24;7(4):1152–61. doi: 10.1021/acsmaterialslett.4c01836 (PMC13730402; doi:10.1021/acsmaterialslett.4c01836)
Supplement: Supplementary file 1 [file tz4c01836_si_007.pdf]

Supporting Information

Freeform Printing Cell-adsorptive Cryogels with Shape-Recoverability

Edgar J. Castanheira[a], Luís P. G. Monteiro[a], Rita Sobreiro-Almeida[a], Vítor M. Gaspar[a], Nina Kølln Wittig[b], Tiago R. Correia[a], Henrik Birkedal[b], João M. M. Rodrigues[a]* and João F. Mano[a]*

[a]CICECO – Aveiro Institute of Materials, Department of Chemistry, University of Aveiro, 3810-193 Aveiro, Portugal
[b]Department of Chemistry and iNANO, Aarhus University, Gustav Wieds vej 14, Aarhus 8000, Denmark

* Correspondence and requests for materials should be addressed to João M. M. Rodrigues (jrodrigues@ua.pt), and João F. Mano (jmano@ua.pt).

General Procedures

Materials and Methods

Gelatin from porcine skin (gel strength 300, type A), Dulbecco's modified Eagle's medium (DMEM) low glucose, formaldehyde, and 6-aminofluorescein were acquired from Sigma-Aldrich. Lithium phenyl(2,4,6-trimethylbenzoyl) phosphinate (LAP) was purchased from Carbosynth. Methacrylic anhydride (>97%), sodium azide (99%) and deuterium oxide (99.8%) were obtained from TCI Chemicals. Triton X-100 and PBS were bought from Merck. hASCs (Homo Sapiens, ATCC PCS-500-011) were purchased from LGC Standards S.L.U. (Barcelona, Spain). Minimum Essential Medium α-modification (α-MEM), fetal bovine serum (FBS, E.U. approved, South American origin), Quant-iT PicoGreen dsDNA Assay Kit, alamarBlue, calcein violet, propidium iodide, and 4',6-diamidino-2-phenylindole (DAPI) were purchased from Alfagene (Lisbon, Portugal). Dulbecco's PBS (dPBS) was purchased from Thermo Fisher Scientific (Oeiras, Portugal). Flash Phalloidin Green 488 was purchased from Biolegend (California, USA). Trypsin/EDTA was purchased from Bioscience Lonza (Gampel, Switzerland). Spectra/Por dialysis membranes with a molecular weight cut-off (MWCO) of 3.5 kDa were supplied from Spectrum Laboratories. ULTRAVIST™ 370 was obtained from Bayer Schering Pharma AG (Berlin, Germany). Unless otherwise specified, all used reagents were of analytical grade and used as received, without further purification.

Synthesis and characterization of gelatin methacryloyl (GelMA)

GelMA was synthesized using a procedure previously reported.[1] Briefly, 2.0 g of gelatin was dissolved in 20.0 mL of PBS. The solution was heated to 50 °C, and 800 µL of methacrylic anhydride was added dropwise. The reaction mixture was stirred at 50 °C for 4 h. The reaction was then neutralized and stopped by adding an equimolar amount of NaOH solution (1M). The modified gelatin solution was purified by dialysis using a membrane with a MWCO of 3.5 kDa for at least 3 days against distilled water, and the water was changed twice a day. Purified GelMA was obtained by freeze drying, giving rise to a white foam (1.8 g, yield 92 %) and stored at 4 °C, protected from light. The chemical modification was assessed by ^{1}H-NMR. ^{1}H NMR spectra were recorded using a Bruker Avance-300 spectrometer (Bruker, Germany) operating at 300.13 MHz, using D_2O. The chemical shifts were expressed in δ (ppm).

Determination of the Degree of Substitution

The insertion of methacryloyl groups onto the gelatin backbone was confirmed and quantified by ^{1}H NMR spectroscopy analyses. The degree of substitution (DS) was calculated based on a

protocol previously reported.[2-3] Briefly, gelatin without functionalization was used to calculate the DS of methacryloyl groups present in GelMA, resulting in a DS of 89 ± 4% according to Equation (SI 1).

$$DS = 1 - \frac{I(LMP\ GelMA)}{I(LMP\ gelatin)} \qquad \text{Equation (SI 1)}$$

Where I(LMP GelMA) and I(LMP gelatin) are the relative integration areas (I) corresponding to lysine methylene protons (LMP) of GelMA and gelatin, respectively, at δ = 2.86 ppm (Figure SI 1). The decrease in the signal in the GelMA spectra in relation to gelatin confirms the modification degree. Since the proton signal of the aromatic moieties in gelatin remained constant, the intensity was used to normalize the intensity of lysine protons. Accordingly, the DS was calculated by comparing the proton signal at δ = 2.86 ppm of unmodified gelatin and GelMA.

Sol-gel transition process

To observe the sol-gel transition, four different solutions were analyzed by ^{1}H-NMR, both before and after 5 min of irradiation with a blue light at 1000 mW/cm^2 (λ = 395-480 nm), at 4 °C and 37 °C (Figure SI 3). At both temperatures the vinyl protons of the methacrylic groups (δ = 5.09 and 5.33 ppm for 4 °C and δ = 5.47 and 5.71 ppm for 37 °C, respectively) disappear. However, at 37 °C no broadening was observed.

Rheological properties

A Kinexus Lab+ (Malvern Panalytical, UK) was used to characterize the rheological properties of the GelMA biomaterial ink at concentrations of 1%, 2% and 3% (w/v). The gap setting was fixed at 0.5 mm, the tests were performed at 25 °C with a frequency of 1.0 Hz, and the shear strain was set between 0.01 – 3% (LVER). The elastic and viscous moduli of the precursor solution during the photopolymerization process were studied using photo-rheology, employing a blue light at 1000 mW/cm^2 (λ = 395-480 nm) at two different temperatures (37 °C and 4 °C). After reaching the gel point, all the following measurements were performed at 4 °C. Additionally, the shear-thinning behavior of the material was investigated by analyzing shear viscosity (Pa.s) as shear stress (Pa) increased. Thixotropy properties were also examined, evaluating the time needed for the microstructural rearrangement occurring in a shear-thinning fluid following an applied shear rate, specifically the extrusion shear rate used during the printing process. To

determine this value, the viscosity was analyzed as the shear rate increased, and the data was fitted to the power law equation (SI 2) to calculate the power law index (n).[4-5]

$$\eta = K\dot{\gamma}^{n-1} \qquad \text{Equation (SI 2)}$$

Where η is the shear viscosity, γ is the shear rate, n is the power law index, and K is the consistency index. From this, we calculate the power law index to calculate the shear rate at the printer nozzle equation (SI 3). $\dot{\gamma}_w$ is the shear rate at the wall of the nozzle, Q the volumetric flow rate through the nozzle, and r is the nozzle inner radius [5].

$$\dot{\gamma}_w = \frac{Q}{\pi r^3}\left[3 + \frac{1}{n}\right] \qquad \text{Equation (SI 3)}$$

The value of shear rate obtained for a printing pressure of 45 kPa at 4 °C was 135.1 s^{-1}.

Biomaterial ink formulation

The biomaterial ink was prepared by dissolving GelMA at concentrations of 1%, 2% and 3% (w/v) in double-distilled and deionized filtered (0.22 µm) (MilliQ) water. To this solution, 0.5% (w/v) of LAP was added, and the solution was allowed to stabilize at 4 °C overnight before use. The biomaterial ink was used at both 4°C and 25°C following a stabilization period for rheological assays. However, extrusion was carried out exclusively at 4°C to ensure consistent filament deposition and maintain uniformity throughout the printing process. Additionally, green food dye was added into the solution for most applications. To prepare the support bath for 3D printing, xanthan gum was dissolved in MilliQ water at a concentration of 5% (w/v), with the addition of 0.05% (w/v) sodium azide to prevent bacterial growth. The support bath was stored in the fridge at 4 °C.

Casted cryogel preparation

The biomaterial ink formulation was transferred into PDMS molds with a 5 mm diameter and 5 mm height. The mold was then frozen at –20 °C overnight for subsequent photopolymerization with blue light (Valo™ grand: λ = 385-515nm; 3 mins of exposure time at 1000 mW/cm^2). Subsequently, the scaffolds were removed from the PDMS mold and allowed to defrost and equilibrate in PBS for 24 h before further tests.

Biomaterial ink preparation and 3D printing

A 2% (w/v) GelMA solution containing 0.5 % (w/v) of LAP was prepared, and the mixture was stirred at 37 °C until complete dissolution was achieved, as mentioned previously in the biomaterial ink formulation. Subsequently, the solution was transferred to a printer's cartridge and stored overnight at 4 °C. A 22G (410 µm inner diameter) blunt needle was used to print the biomaterial ink into a 5% (w/v) xanthan gum supporting bath. All printing models were designed using Shapr3D software (version 5.450) and post-processed by the BioX HeartOS (V2.0, CELLINK) using a BioX bioprinter (BioX, CELLINK) to fabricate the 3D models. Both the printing head and print bed were set at 4 °C during the printing process. The printing pressure was adjusted in increments of 10 kPa, ranging from 55 kPa to 75 kPa, while three different printing speeds were tested: 4 mm/s, 7 mm/s, and 10 mm/s. This variation was aimed at identifying the optimal printing conditions to achieve high filament fidelity and ensure the precision of the printed structures. After printing, the scaffolds were frozen overnight at -20 °C while immersed in the xanthan gum bath. Afterwards, the structures were irradiated for 3 min with blue light (395-480 nm, Valo™ grand at 1000 mW/cm^2). Lastly, to clean the printed structures, the xanthan gum bath with the irradiated scaffolds was immersed in water and placed in a shaking bath at 37°C until the scaffolds were visually free of any traces of xanthan gum. On the next day, the water was carefully removed, and the structures were washed twice with distilled water. After the washing, the samples were stored in PBS at 4°C until further use.

Evaluation of the shape fidelity of the printed scaffolds

The filament fidelity (filament diameter) was assessed by printing filaments with 1 cm length while varying printing speed from 4 to 10 mm/s and the pressure from 55 to 75 kPa. The measurements were converted into "filament printability" by dividing the filament diameter by the nozzle gouge (410 µm), and an acceptable filament fidelity region was established between 0.85 and 1.15 (349 and 472 µm). Next, a 2 cm x 2 cm square mesh (total size) was printed to access the total area of the construct, shape fidelity, and mesh angle. To evaluate the shape fidelity (printability factor – *Pr*) of the printed square meshes, a previously reported methodology was used based on equation (SI 4): [6]

$$Pr = \frac{L^2}{16A} \qquad \text{Equation (SI 4)}$$

Where Pr is the printability factor (circularity), A is the single mesh area, and L the perimeter of a single mesh.

Mechanical analysis

Using a Universal Mechanical Testing Machine, Shimadzu MMT-101N (Shimadzu Scientific Instruments, Kyoto, Japan), equipped with a load cell of 100 N, the mechanical behavior of P-cryogels and C-cryogels was evaluated with compression tests. Both unidirectional and cyclic compression assays were performed at room temperature on freshly hydrated cylindrical specimens with a diameter of approximately 5.0 mm and a height of 5.0 mm at a constant rate of 3 mm/min. Young's modulus was defined as the slope of the linear region of the strain–stress curve, corresponding to a maximum of 10% strain. The recovery percentage was calculated by taking the compressive stress (kPa) of the first cycle as 100% for the dynamic strain-stress behavior. The 10-cycles dynamic strain-stress test was performed at 40% and 80% compressive strain. Toughness was calculated as the area under the stress-strain curves. The dissipation of energy was calculated by the hysteresis loop, represented by the area enclosed within the hysteresis loop (loading and unloading strain-stress curves) as the amount of mechanical dissipated energy.[7]

Scanning electron microscopy (SEM)

SEM was used to evaluate the morphology of the scaffolds, including rugosity, pore size, and interconnectivity. For this purpose, C-cryogel and P-cryogel were sliced into thin sections using a scalpel (approximately 2 x 2 mm^2; diameter x height). Next, the specimens were deposited on a metal stub containing an O.C.T. compound (Tissue-Tek, Sakura, Japan) and analyzed in a SU-3800 (Hitachi, Japan) at -15 °C. The system is coupled with a standard SEM cooling stage ranging from -25 °C to 50 °C (Deben, UK). Pore size analysis was performed using ImageJ software (LOCI, University of Wisconsin) with a defined pore size interval between 1 to 1000 µm of diameter and with a minimum sphericity of 0.1.

For the degradability assays, the samples were analyzed in their intact form as retrieved from the assay, to avoid potential damage caused by slicing that could lead to inaccurate conclusions. The only additional step was carefully washing the cryogels with Milli-Q water and gently patting them with Kimwipes to remove excess surface salts. Furthermore, the pore size was manually and meticulously measured (n=40) for day 0 and day 14 for the following formulations: C-cryogels, P-cryogels, and injected P-cryogels.

Water uptake

To evaluate the swelling ratio of cryogels and freeze-dried scaffolds (n=6), freshly prepared specimens were immersed in PBS for 15 min and 24 h. The swelling ratio (Qm) was calculated using equation (SI 5):

$$Qm = \frac{ms}{md} \times 100$$ Equation (SI 5)

Where **ms** and **md** are the weight of hydrated and dried gels, respectively. Pore connectivity was assessed using a water-wicking technique, as previously reported.[8] Briefly, interconnected porosity was calculated as the leaked volume over the total volume. The leaked volume is defined as the water mass wicked away using Kimwipe paper after the specimens were soaked in water for 1 h (total volume).

X-ray microtomography

DP-cryogel and HP-cryogel were analyzed by X-ray microcomputed tomography (µCT) using a Zeiss Xradia 620 VERSA X-ray microscope (Carl Zeiss Microscopy GmbH, Jena, Germany) at the Aarhus X-ray imaging alliance (AXIA) infrastructure with an X-ray voltage of 40 kV and operated at 3W. DP-cryogel was fixed in a sealed plastic sample holder to prevent exposure to air. The DP-cryogel was then hydrated (HP-cryogel) and immersed in an iodine ULTRAVIST® 370 solution (diluted 1:10 in water) for a day. Afterward, it was patted with Kimwipe paper, followed by immersion in mineral oil for measurement.

For each tomogram, 3201 projections/360° were recorded using a 4.0x objective with an exposure time of 4 s, to result in a reconstructed isotropic voxel size of 2.5 µm. An LE1 filter was used to reduce the effects of artifacts and to enhance the contrast between the air and the sample. The reconstruction was performed with software provided with the X-ray microscope using a scaling window of the 2D projections that was adjusted to include all features with relevant intensities. Thickness mesh, interconnected porosity, and isolated pores analysis were all visualized and calculated using Dragonfly (ORS, Montréal, Canada). To compare DP-cryogel and HP-cryogel, a virtual cylinder with dimensions of 3 x 3.8 mm^2 (height x diameter) was cropped from the sample centers for analysis to avoid including air surrounding the samples in the calculated percentages of isolated pores and interconnected porosity. Interconnected porosity was calculated from the relation between the total volume of air fraction and the total volume from the isolated pores.

Degradability assays

C-cryogels and P-cryogels were prepared as described in the sections “Casted Cryogel Preparation” and “Biomaterial Ink Preparation and 3D Printing,” respectively. For this assay, P-cryogels were printed into cubic geometries (2.5 mm × 2.5 mm × 2.5 mm), while C-cryogels were cast in a PDMS mold with a volume of 15 µL to ensure that both cryogels types had similar

dimensions. All cryogels were weighed on day 0, with half of the P-cryogels also injected through an 18 G syringe. C-cryogels were not injected, as this level of compression would lead to their disintegration. After weighing, all cryogels were then incubated in 1 mL of PBS at 37 °C for 14 days. Following the incubation period, the cryogels were carefully washed with Milli-Q water, gently blotted with Kimwipes paper, and reweighed. The weight loss was calculated based on the initial weight of the cryogels, accounting for any salt absorption from the PBS solution. To further evaluate the degradability and the effect of injection on the microstructure, all cryogel formulations from both day 0 and day 14 were analyzed using SEM equipped with a cooling stage.

Cell culture

hASCs (ATCC® PCS-500-011™) were used for the viability assays. Cells were initially seeded in a T-175 cm^2 tissue culture flask (Sarstedt) using α-MEM supplemented with sodium bicarbonate (2.2 g/L), 10% heat-inactivated FBS, and 1% antibiotic/antimycotic, and cultured under a controlled atmosphere of 5% CO_2 at 37 °C. Upon reaching confluence, cells were trypsinized and transferred to an ultra-low adhesion 24-well plate (250 x 10^3 hACS per well) with 100 µL of culture medium. The scaffolds were then brought in contact with the cells for 5 minutes and afterward transferred to a different well with fresh medium, where they were cultured for up to 14 days.

Flow cytometry – cell-adsorption

To evaluate the percentage of cells adsorbed by the scaffold hydration, hASCs (80 µL α-MEM, 20,000 cells, n=6) were counted and adjusted to a maximum of 50 µL for analysis. These values were used as the control for the number of cells per well, using forward and side scatter detectors. Subsequently, the dry scaffold was placed in contact with unused wells containing hASCs (80 µL α-MEM, 20,000 cells, n=6). The reminiscent medium was diluted back to 80 µL with fresh α-MEM and used to run the cytometer to count the cells, adjusting to a maximum of 50 µL of sample analyzed. The dilutions were then used to calculate the percentage of total cells that were adsorbed. Data was obtained using a BD AccuriTM C6 Plus flow cytometer, and the subsequent analysis was performed using FlowJo software (Treestar, Ashland, OR, trial version).

Cell morphology and viability

The cell viability (LIVE/DEAD staining) was assessed after 1, 3, 7, and 14 days of culture. At these time points, the cryogel cells were incubated with Calcein-violet/propidium iodide (PI) (Live-dead Kit, Thermo Fischer Scientific) for 20 min, according to the manufacturer's protocol. Additionally, for the cryogel structure staining, the cryogel was incubated an additional 20

minutes before the live-dead kit with 5 mM of 6-aminofluorescein. For F-actin filaments and nucleus staining, the cryogels containing cells were washed with PBS and fixed in 4% (w/v) formaldehyde (Sigma-Aldrich, USA) for 20 min at room temperature. Before staining, the cells were permeabilized for 5 min with a 0.1% solution of Triton X-100. After washing three times with PBS, samples were first incubated in a phalloidin solution (Flash Phalloidin Green 488) diluted 1:40 in PBS at room temperature for 90 min. After washing once with PBS, a DAPI solution (5 µg/mL) diluted 1:200 in PBS was prepared and used to incubate the hydrogels for 5 min at room temperature. After several PBS washes, samples were visualized with a confocal laser scanning microscope (CLSM 900, Carl Zeiss Microscopy, Germany) equipped with a 10x objective. Cryogel structure without cells was also visualized through CLSM by immersing the dried scaffold in a Nile red water solution (0.5mg/mL) for 1 h and then washing it three times with distilled water. The acquired data was processed in Zeiss ZEN v2.3 blue edition software. Cell retention was defined as the quotient between cell viability before and after injection. A bulk star-shaped cell-laden cryogel of larger dimensions (10 x 10 x 1 mm^3; length x width x height) was also visualized under confocal microscopy before and after injection for LIVE/DEAD staining.

Spheroids generation

The MC3T3-E1 cell line (ATCC® CRL-2593) was cultured in DMEM low glucose supplemented with sodium bicarbonate (3.7 g/L), 10% heat-inactivated FBS, and 1% antibiotic/antimycotic, under a controlled atmosphere of 5% CO_2 at 37 °C. Upon reaching confluence, cells were detached with 0.25% trypsin/EDTA and resuspended in their culture medium. A cell density of $3x10^6$ in culture medium (2 mL) was seeded onto AggreWell™ 400 24-well plate (Stem cell technologies, USA) to generate spheroids. Seeded cells were centrifuged at 100 × g for 3 min and incubated for 48 h under standard culture conditions. Spheroids were then rinsed with warm medium and collected in a petri dish using a 37 µm reversible strainer. The scaffolds were slowly brought into contact with 250 µL of medium containing approximately 10 spheroids, and their capability to adsorb these 3D cell agglomerates was evaluated visually.

DNA quantification

Total dsDNA quantification of hASCs was performed using the Quant-iT PicoGreen assay kit (Alfagene, Lisbon, Portugal). Briefly, cryogels were transferred to an Eppendorf and suspended in ultrapure sterile water (250 µL per sample) with 0.1% (w/v) Triton X-100 for cell lysis. After 30 minutes at 37 °C in a shaking water bath, samples were frozen at -80 °C overnight. Subsequently, samples were defrosted, and the supernatant was used according to the specifications of the

manufacturers. A standard curve for DNA analysis was generated with the provided dsDNA solution. After 5 min of incubation at RT, fluorescence was read at an excitation wavelength of 485/20 nm and emission wavelength of 528/20 nm using a microplate reader (Gen 5, Synergy HTX, Biotek).

Cell metabolic activity quantification

To evaluate hASCs cell metabolic activity, alamarBlue™ Cell Viability Reagent (Alfagene, Lisbon, Portugal) was used. Briefly, the cell culture medium was removed, and 10% (v/v) solution of Alamar in α-MEM was added and left to react for 4 h at 37 °C. The results were obtained by measuring the absorbance at λ= excitation/emission - 570/585 nm (Microplate Reader—Synergy HTX with luminescence, fluorescence, and absorbance, Biotek Instruments, Winooski, USA). For the metabolic activity after injection, four formulations were used: two different syringe gauges (12 and 16 G) and two different squared cell-laden cryogels (hollow and bulk – 5 x 5 x 1 mm^3; length x width x height). To assure minimal cell density variation, two metabolic assays were performed back-to-back, before and after injection. The gels were washed five times with dPBS and then cultured again with a 10% (v/v) solution of Alamar in α-MEM. For the control, cryogels that were not injected were also washed and incubated again to check for changes in the metabolic activity. The medium used during the injection was centrifuged, concentrated, and measured by adding Alamar reagent to achieve a 10% (v/v) solution.

Statistical analysis

All data were statistically analyzed using GraphPad Prism 8 software using one-way analysis of variance (ANOVA) and expressed as a mean ± SD (n≥3). To compare filament printability values among different pressures, normality was checked using the Shapiro-Wilk test. Subsequently, a Kruskal-Wallis test followed by Dunn's *post hoc* test was employed. The differences were considered significant for a *p*-value < 0.05. Only significant differences are presented for simplicity in data analysis.

Supplementary Figures

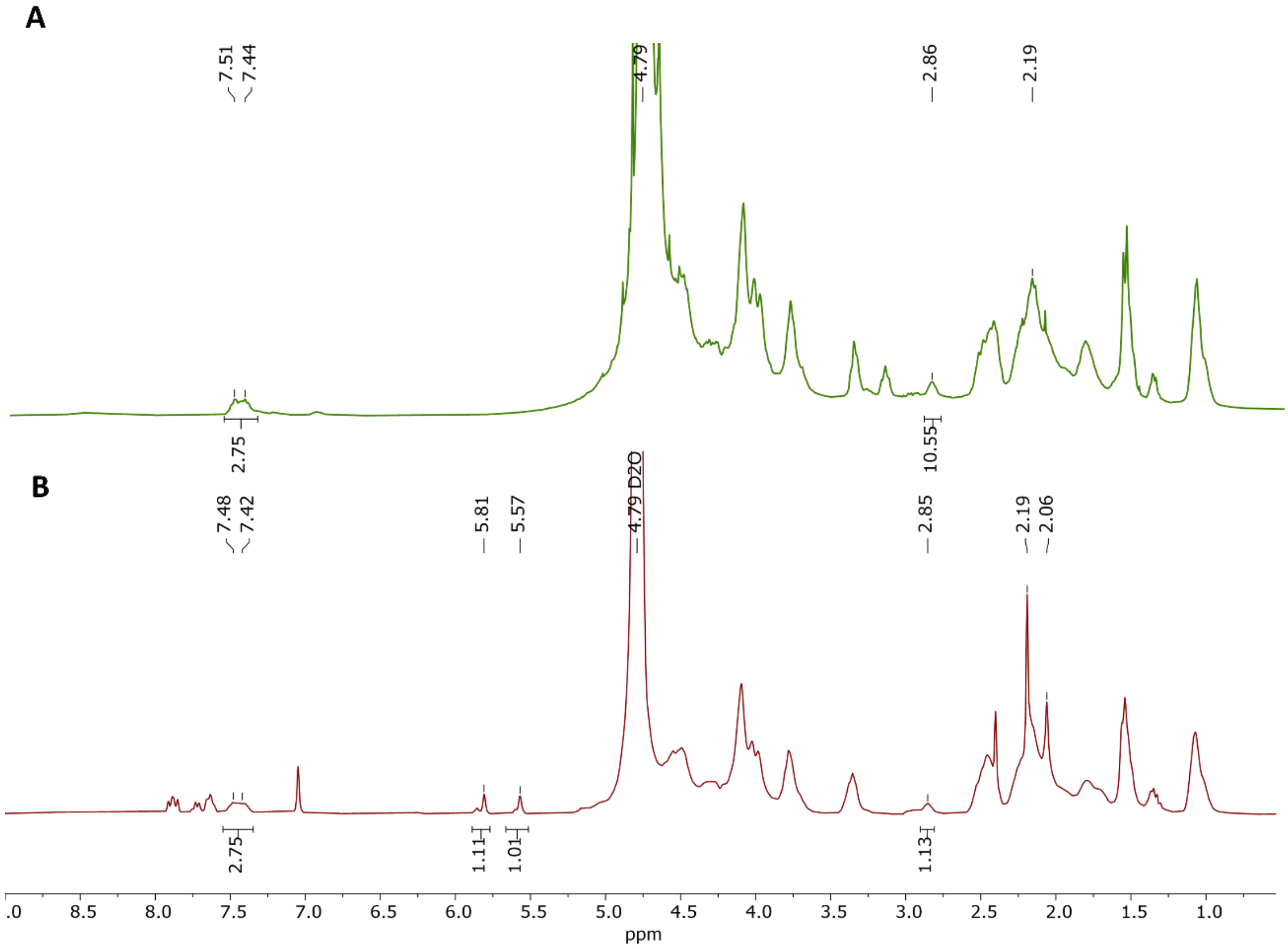

Figure SI 1 – ^{1}H NMR spectra of **a)** gelatin and **b)** GelMA, in D_2O, used to calculate the DS.

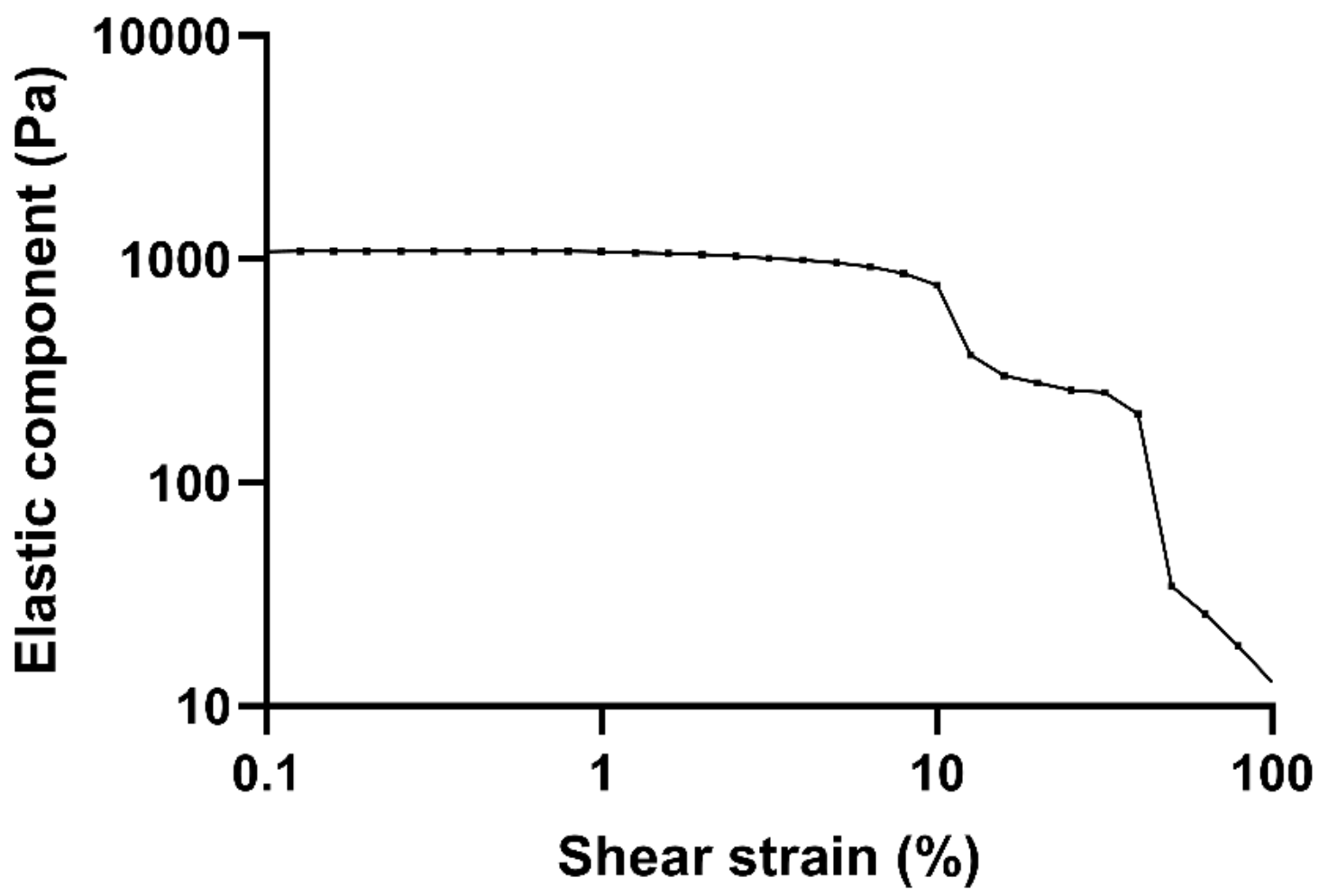

Figure SI 2 – Determination of the linear viscoelastic region (LVER) for the biomaterial ink of gelatin at 37 °C.

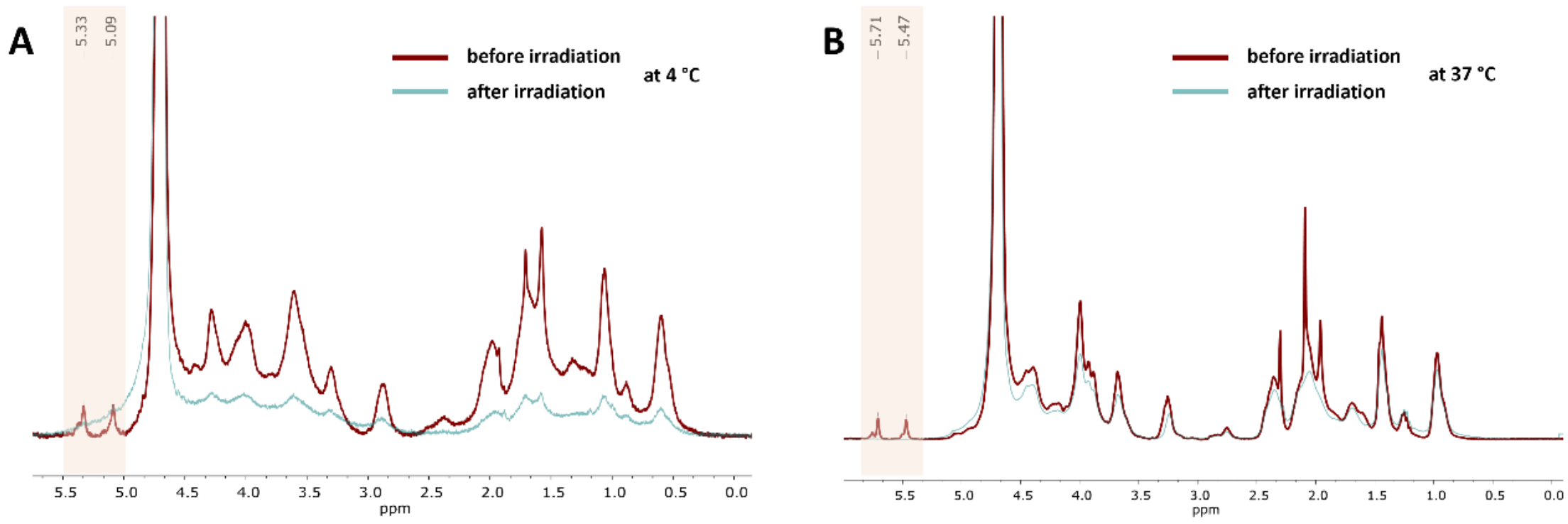

Figure SI 3 – ^{1}H NMR spectra of GelMA in D_2O before and after irradiation. **a)** at 4°C **b)** 37°C.

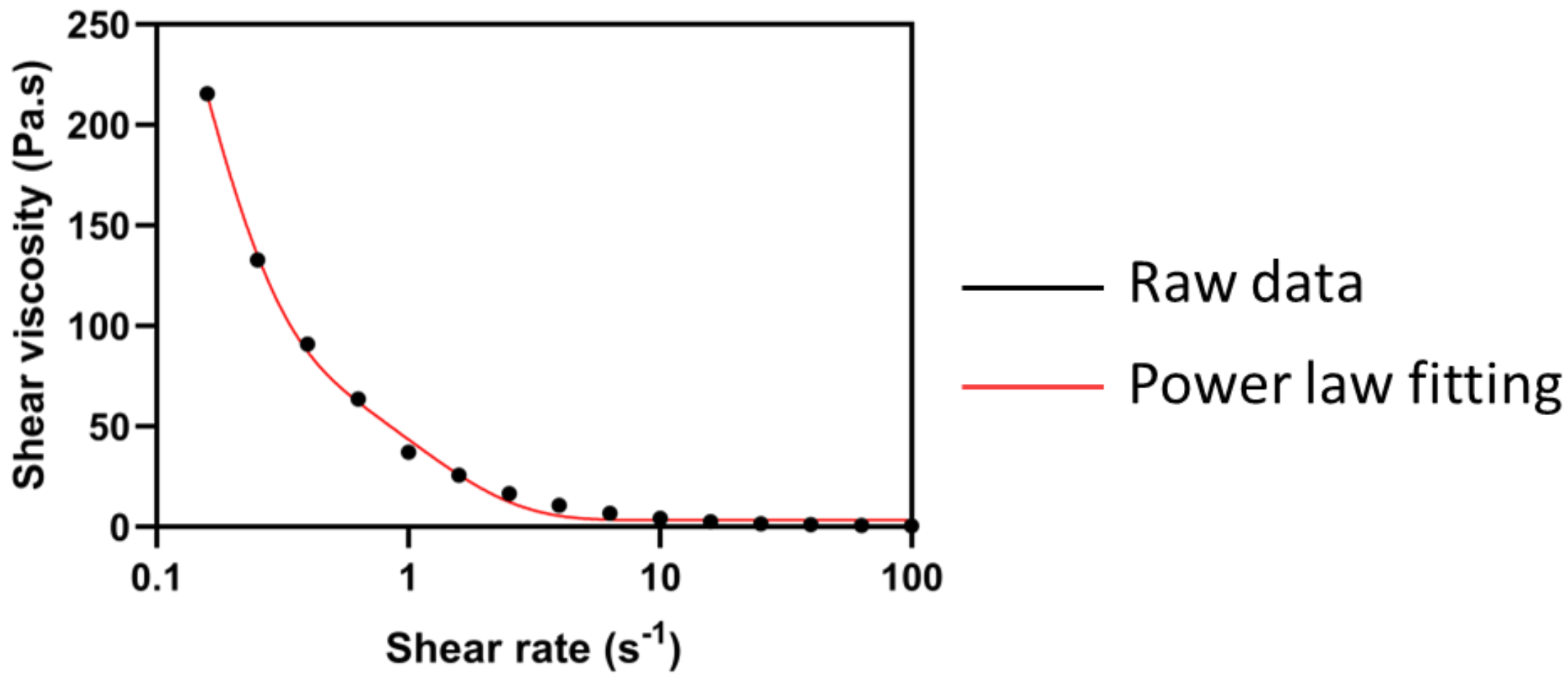

Consistency index	Correlation coefficient
38,13	0,9986
24,47	0,9977

Figure SI 4 – Rheological assessment of the table of shear rates for 2% (w/v) GelMA ink. Power law fitting for the data achieved.

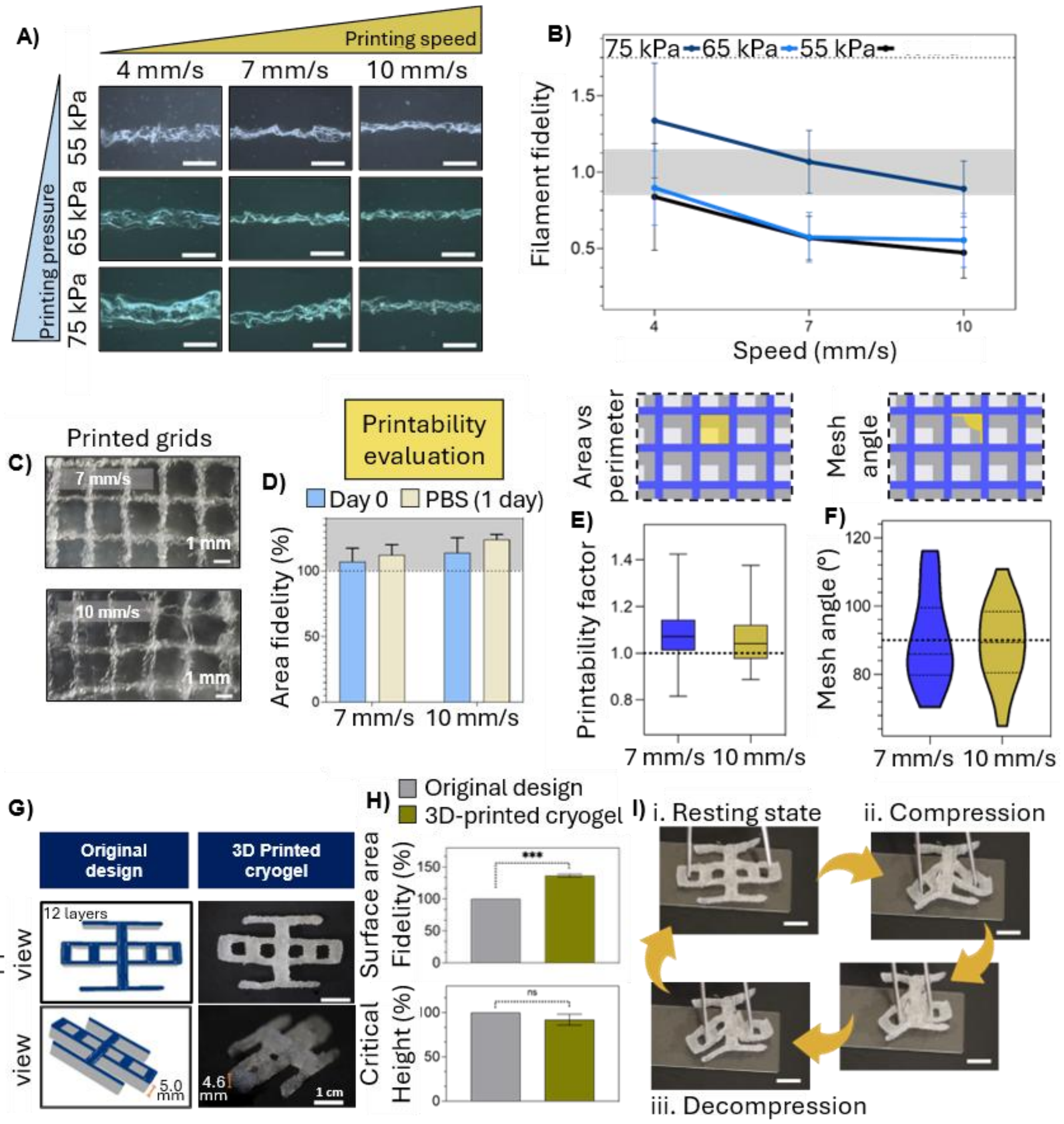

Figure SI 5 – Optimization of the freeform 3D-printed conditions. A) Filament extrusion of GelMA 2% (w/v) at 4 °C using different speeds and pressures; Scale bar = 1 mm. B) Corresponding comparison between the inner diameter of the syringe gauge and the actual printed filament (filament fidelity). C) Photographs of the square mesh printed structures using the pressure (75 kPa) and speed values (7 or 10 mm/s) with the best filament fidelity. Evaluation of the printed grids parameters: D) Area fidelity (before and after being immersed in PBS), E) Printability (*Pr.*), and F) mesh angle. G) Shape complexity and height fidelity (printed vs stereolithographic). H) Critical height and surface area fidelity of the respective printed complex structure. I) Structural integrity of 3D-printed scaffold during compression and decompression; Scale bar = 1 cm.

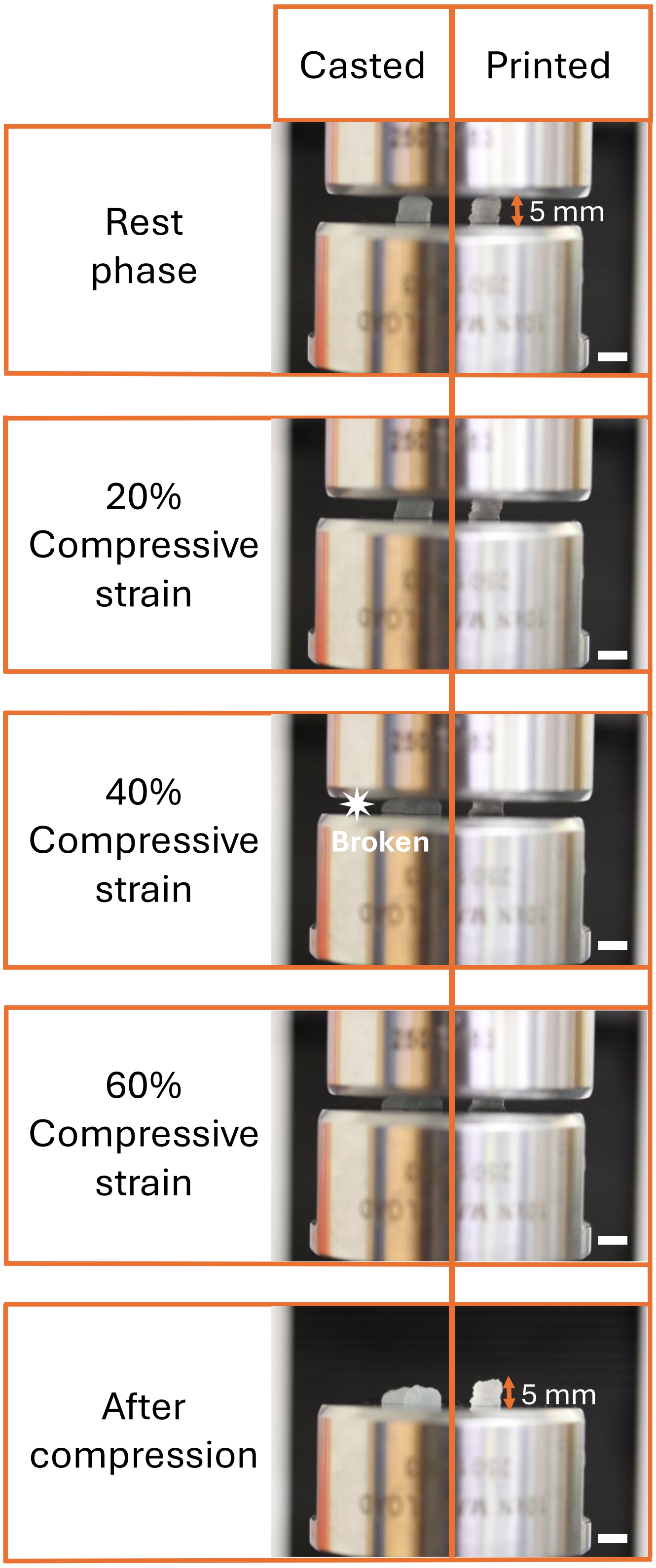

Figure SI 6 – Comparative analysis of the full compression cycle, from the resting phase to 60% compressive strain and back to the resting state, in 20% strain intervals, for both casted and printed cryogels. The breaking point of the casted cryogels and the recovery behavior of the printed cryogels are highlighted. Scale bar = 5 mm.

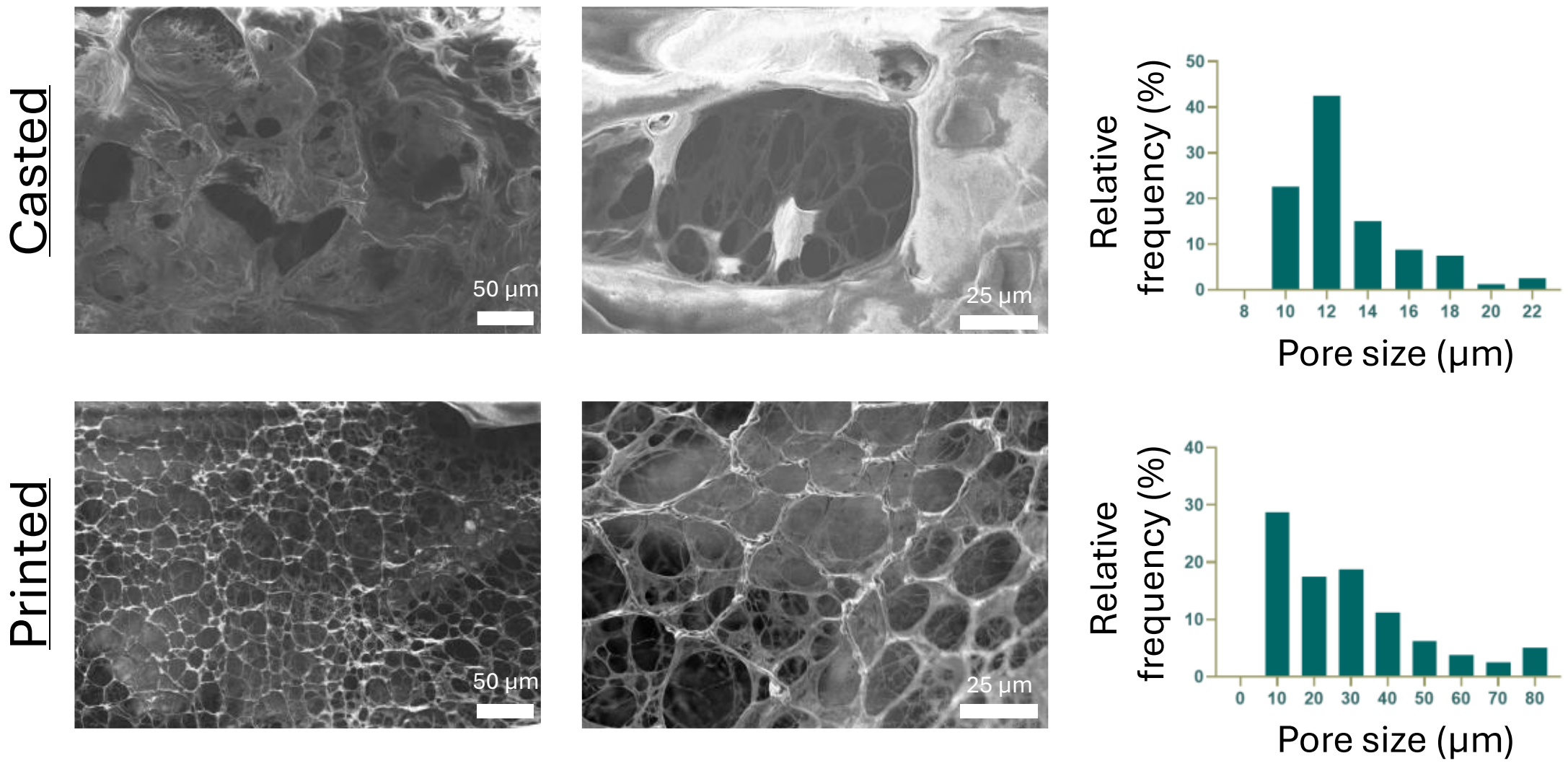

Figure SI 7 – Microporosity analysis of casted and printed cryogels using SEM with a cooling stage. Images at two different magnifications (left) highlight the porosity in both fabrication methods. The frequency distribution of microporosity for each cryogel type is shown (right).

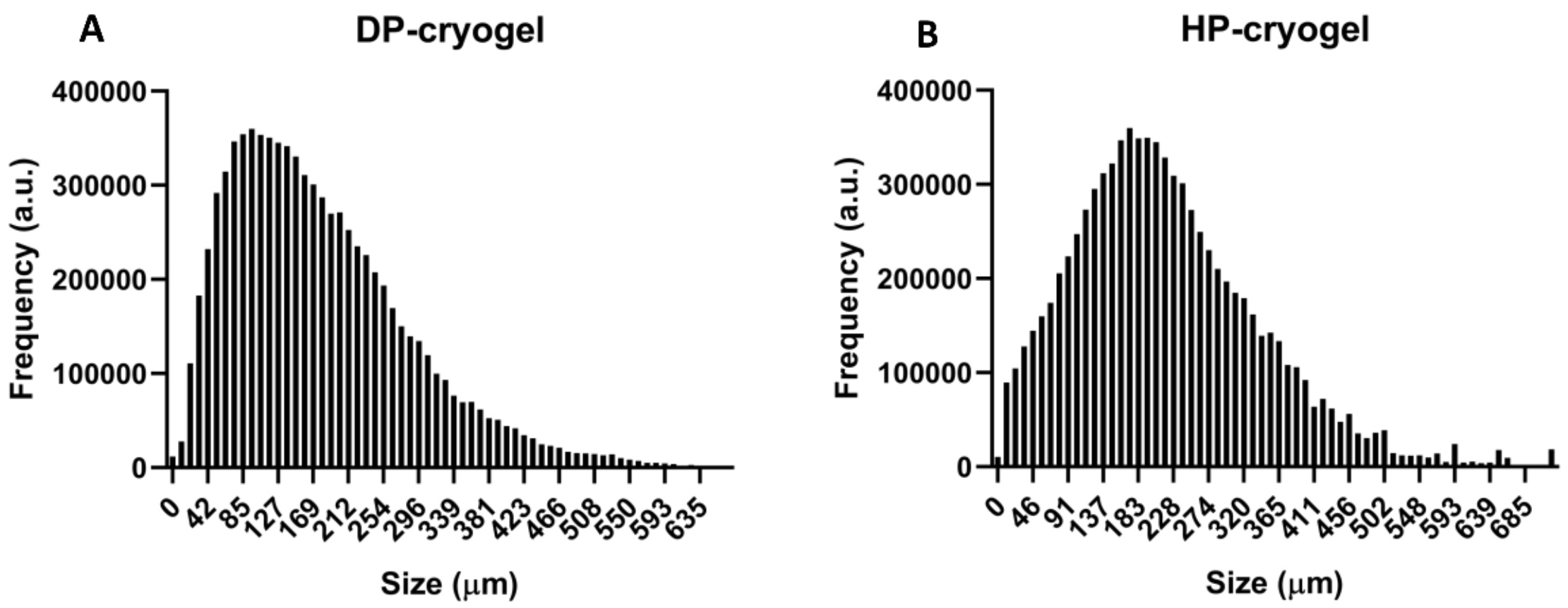

Figure SI 8 – Pore size histograms assessed by X-ray micro-computed tomography of **a)** Dried printed (DP)-cryogel and **b)** Hydrated printed (HP)-cryogel.

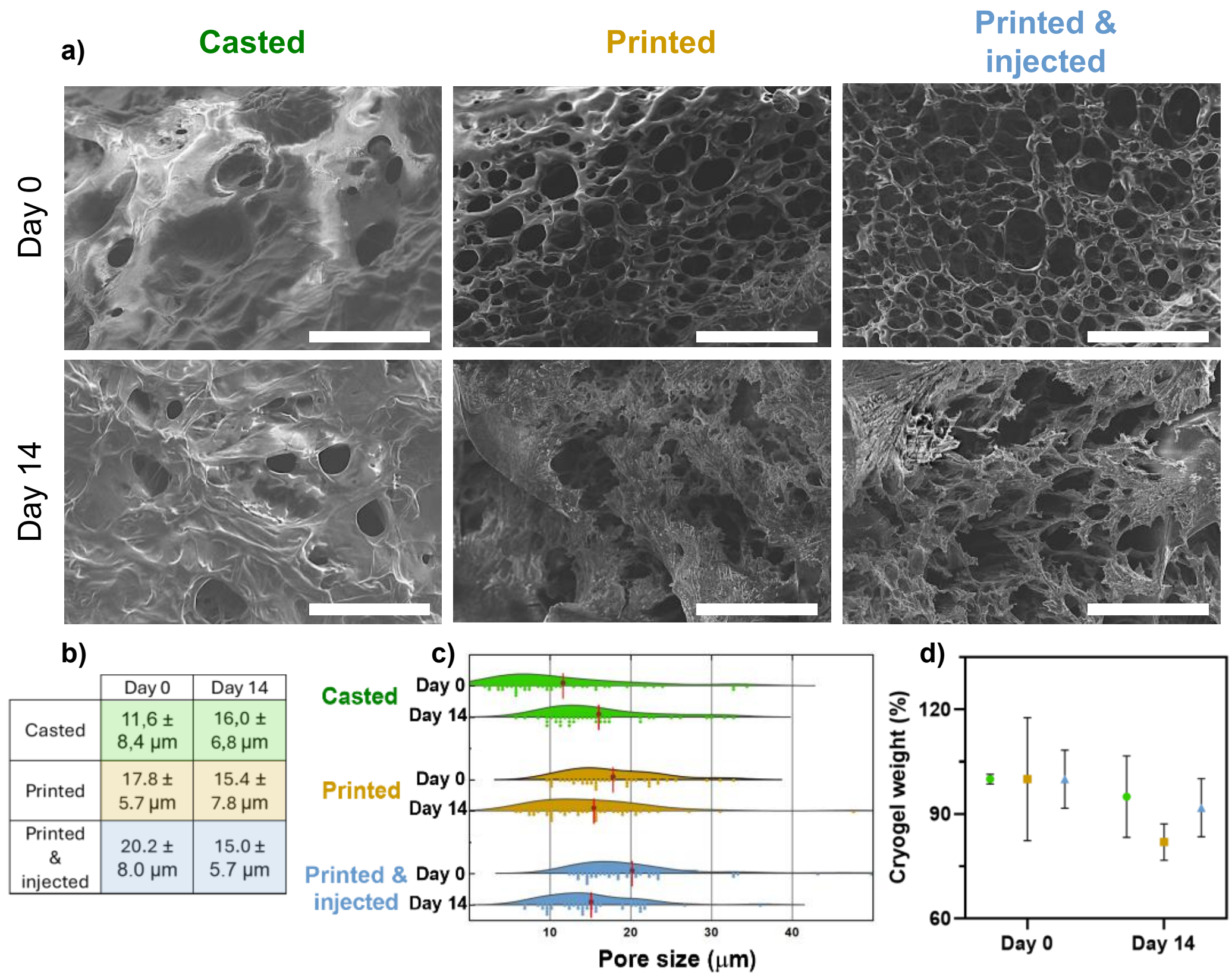

Figure SI 9 – Structural and degradability analysis of P-cryogels and C-cryogels. (a) SEM images of C-cryogels and P-cryogels without being injected and after injection at 65% compression. The same type of cryogels were also observed after 14 days of incubation at 37 °C in PBS. Scale bar = 100 µm. (b) Quantitative analysis of pore size in C-cryogels, P-cryogels without injection, and P-cryogels with injection at day 0 and after 14 days of incubation at 37 °C in PBS. (c) Comparison of pore size changes between C-cryogels, P-cryogels without injection, and P-cryogels with injection at day 0 and after 14 days of incubation at 37 °C in PBS. (d) Weight loss measurements of C-cryogels, P-cryogels without injection, and P-cryogels with injection at day 0 and after 14 days of incubation at 37 °C in PBS.

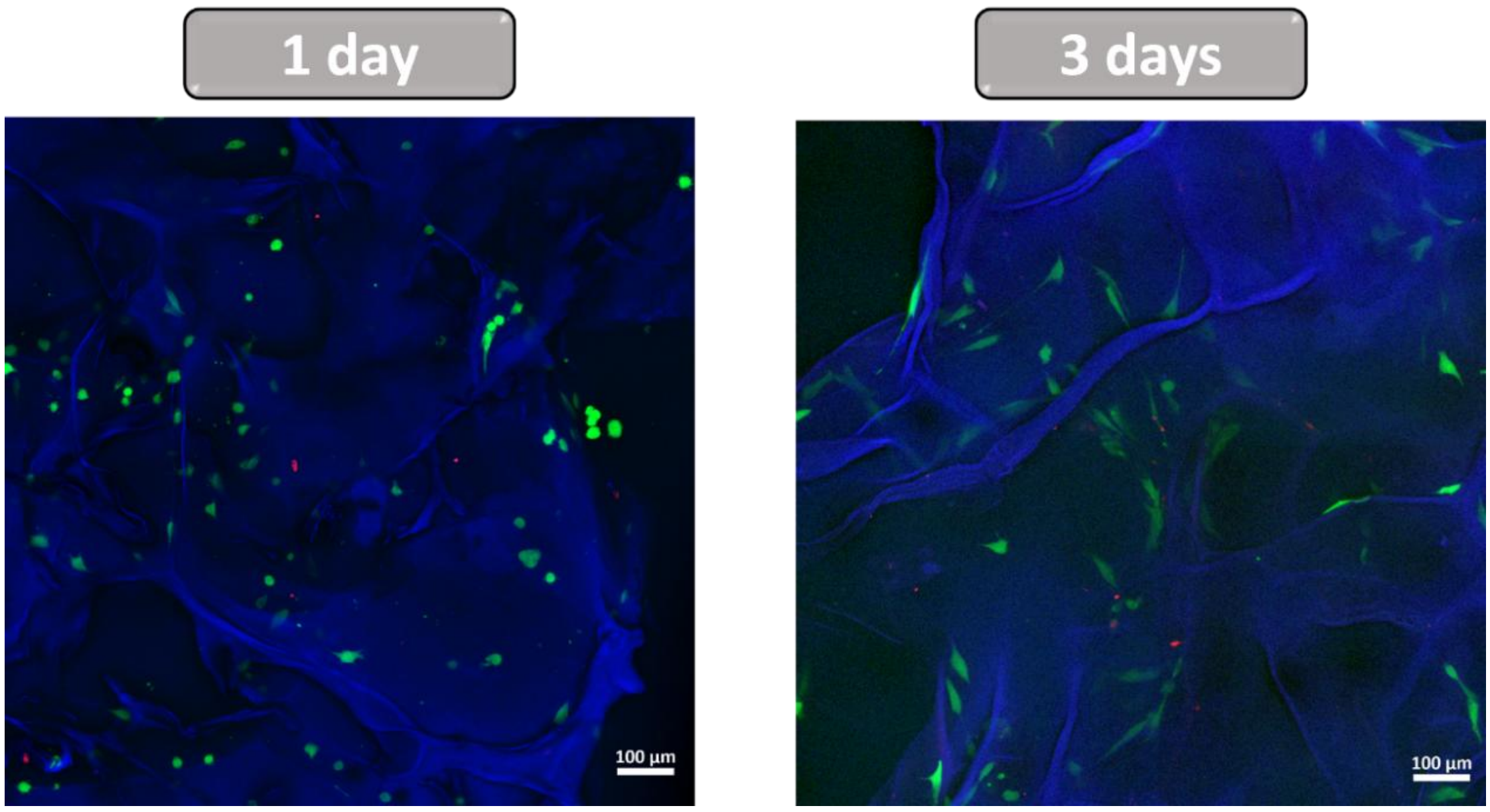

Figure SI 10 – Live-dead images (green: Calcein AM; red: PI) of the printed GelMA cryogel incubated with human adipose-derived stem cells (hASCs) after 1 and 3 days of culture.

References

1. An I. Van Den Bulcke, B. B., Nadine De Rooze, Etienne H. Schacht, Maria Cornelissen, and Hugo Berghmans, Structural and Rheological Properties of Methacrylamide Modified Gelatin Hydrogels. *Biomacromolecules* **2000,** *1*, 31-38.
2. Zhu, M.; Wang, Y.; Ferracci, G.; Zheng, J.; Cho, N. J.; Lee, B. H., Gelatin methacryloyl and its hydrogels with an exceptional degree of controllability and batch-to-batch consistency. *Sci Rep* **2019,** *9* (1), 6863.
3. Li, X.; Chen, S.; Li, J.; Wang, X.; Zhang, J.; Kawazoe, N.; Chen, G., 3D Culture of Chondrocytes in Gelatin Hydrogels with Different Stiffness. *Polymers (Basel)* **2016,** *8* (8).
4. Nair, K.; Gandhi, M.; Khalil, S.; Yan, K. C.; Marcolongo, M.; Barbee, K.; Sun, W., Characterization of cell viability during bioprinting processes. *Biotechnol J* **2009,** *4* (8), 1168-77.
5. Lopez Hernandez, H.; Souza, J. W.; Appel, E. A., A Quantitative Description for Designing the Extrudability of Shear-Thinning Physical Hydrogels. *Macromol Biosci* **2021,** *21* (2), e2000295.
6. Ouyang, L.; Armstrong, J. P. K.; Lin, Y.; Wojciechowski, J. P.; Lee-Reeves, C.; Hachim, D.; Zhou, K.; Burdick, J. A.; Stevens, M. M., Expanding and optimizing 3D bioprinting capabilities using complementary network bioinks. *Science Advances 6* (38), eabc5529.
7. Basu, S.; Johl, R.; Pacelli, S.; Gehrke, S.; Paul, A., Fabricating Tough Interpenetrating Network Cryogels with DNA as the Primary Network for Biomedical Applications. *ACS Macro Letters* **2020,** *9* (9), 1230-1236.
8. Bencherif, S. A.; Sands, R. W.; Bhatta, D.; Arany, P.; Verbeke, C. S.; Edwards, D. A.; Mooney, D. J., Injectable preformed scaffolds with shape-memory properties. *Proc Natl Acad Sci U S A* **2012,** *109* (48), 19590-5.
